# Layer-specific genetic variation unlocks secondary metabolite diversity in long-lived clonal peppermint

**DOI:** 10.1073/pnas.2532794123

**Published:** 2026-05-08

**Authors:** Nestor Kippes, Meric C. Lieberman, Darrin Culp, Isabelle J. DeMarco, Helen T. Tsai, Kanae Masuda, Niccolò Terzaroli, Jordan Lopez, Robert G. Wilson, Luca Comai, Isabelle M. Henry

**Affiliations:** ^a^Department of Plant Biology and Genome Center, University of California at Davis, Davis, CA 95616; ^b^University of California Cooperative Extension, Agriculture and Natural Resources, Intermountain Research and Extension Center, Tulelake, CA 96134; ^c^Department of Agricultural, Food, and Environmental Sciences, University of Perugia, Perugia 06121, Italy; ^d^Mars Wrigley, Ingredient Science, Chicago, IL 60642; ^e^Department of Botany, Chulalongkorn University, Bangkok 10330, Thailand

**Keywords:** chimera, breeding, meristematic layer, γ-mutagenesis, peppermint

## Abstract

Mutations that arise in the shoot apical meristems can become fixed, but typically only in one of the meristem layers. Therefore, in long-lived, clonally propagated species, polymorphic genomes coexist in the form of periclinal chimeras. Given their evolutionary and economic impact, it is critical to understand the dynamics and phenotypic implications of layer-specific variation. Here, we γ-irradiated axillary buds from an elite peppermint clone and obtained 261 independent mutants carrying large indels. We produced a haplotype-aware, high-continuity assembly of this sterile allohexaploid and, using short-read sequencing, detected, on average, six large indels per mutant. Importantly, most of these mutants were periclinal chimeras: comparison of mutation frequency in root (derived solely from the L2/3 layer) and leaves (which contain cells from all three layers) demonstrated that the indels are confined to either the outer, L1-derived layer, or the inner L2/3 layers. We observed that the L1 layer was more often mutated, confirming that mutation rate in the shoot apical meristem is potentially optimized to each meristematic layer. To assess whether deletion of a single haplotype in a single meristematic layer could affect plant function, we characterized mutants under field conditions, detecting variation in secondary metabolite production. Two mutants produced an oil with very low (−)-menthol levels, associated with the loss of a single haplotype of the menthone-menthol reductase gene in the epidermal layer. These results highlight the evolutionary relevance of layer-specific genetic variation and present opportunities for improvement of clonally propagated crops that suffer from genetic diversity bottlenecks.

## Significance

Mutations arise randomly during a plant’s life cycle. Most often they are transient, unless they occur in founder cells, such as stem cells and gametes. In long-lived, vegetatively propagated organisms, a stem cell mutation may be confined to a single layer, such as the epidermis, forming a periclinal chimera. Here, we induced genetic variation in peppermint using ionizing radiation and show that most mutants are chimeric. The identification of stable layer-specific mutants highlights the potential impact of epidermal chimeras on plant fitness. Furthermore, mutations that affected only the epidermis resulted in profound changes in oil profiles, highlighting the opportunities associated with layer-specific trait improvement in clonally propagated plants.

Mutations can arise at any time during a plant life cycle but unless they occur in founder cells, such as gamete, zygote, or meristem stem cells, they are rapidly lost. Founder cell mutations can be transmitted to the next sexual generation. Stem cells, however, have specific fates according to the meristem layer (L) in which they occur. L1 stem cells produce the epidermis, L2 the parenchyma and the germline, and L3 the ground tissue including the adventitious vascular system. Therefore, some organs, such as trichomes (L1) or roots (L3) originate from a single cell layer while others (leaves for example) originate from all three.

Stem cell mutations can persist and form periclinal chimeras, in which a mutation is homogeneous within the meristematic cell layer in which it appeared, but absent from the other layers. In fact, most fixed somatic mutations in plants are layer-specific (1, 2), generating distinct clonal variants (3–7) in the form of periclinal chimeras. A periclinal chimera may provide selective advantages, for example if a mutation is detrimental when present in all plant tissues, but beneficial when present in a single layer. The L1-derived epidermis, for example, interacts with biological and physical agents and is the site of production of secondary metabolites, such as essential oils, that mediate defenses against herbivores. Documented examples, however, are limited. Layer-specific mutations can also result in new commercially valuable traits (7, 8), as exemplified in potato (9–13), orange (14), and grapes (15–19). In long-lived plants and clonally propagated species, such layer-specific mutations can be highly stable, as for example in grapevine Pinot Meunier (20). Layer-specific genetic variation is also critical to transformation and regeneration efforts. For example, a recent effort to introduce resistance to Cassava brown streak disease (CBSD) in cassava, through transformation and in-vitro regeneration, resulted in acquired resistance to CBSD but novel sensitivity to Cassava mosaic disease (CMD) (21). This loss of resistance to CMD was later attributed to periclinal chimerism of the original plant and absence of the resistance allele in the meristematic layer that produced the new regenerant (22). It is not clear, however, whether important phenotypes, such as oil composition, can be influenced by genetic variants that are confined to one specific tissue. Given their evolutionary and economic impact, it is critical to understand the effect of layer-specific genotypic variation, especially in the context of long-lived asexually propagated plants.

Studies in apricot have demonstrated that most somatic mutations are layer-specific. Furthermore, the authors observed a bias in mutation rates, with the L1 layer exhibiting a higher (1.8x) number of mutations than the L2/3 layer (1). Recent work in two different potato varieties also indicated that the mutation rate in the L1 layer is higher than in the L2/3 layer (2). Together, these findings suggest that mutation rates within the shoot apical meristem are perhaps optimized according to the different developmental fates of meristematic cells: L1 progenitor cells might accumulate mutations faster in order to provide useful variation with little long-term genetic consequences, while L2 progenitor cells might be protected because they will produce the germline. In other words, these results suggest that the layered organization of angiosperm meristems evolved to balance genetic fidelity with adaptability. The mechanisms underlying the mutation rate differences between layers remain to be deciphered. One possibility is that the L1 layer is more prone to mutations because it is more exposed. Here, we tested this hypothesis by documenting whether a bias in layer mutation rate is also visible following induced γ-irradiation mutagenesis in peppermint. γ-irradiation is a penetrating mutagen that is not expected to affect the layers differently.

Mint (genus Mentha) is a clonally propagated specialty crop and a versatile aromatic plant widely valued for its culinary, medicinal, and flavorful properties, and enjoying significant popularity in contemporary cultural practices. Like most Lamiaceae, Mentha species synthesize defense oils in epidermal protrusions called glandular trichomes (23). These oils have commercial importance. Peppermint (*Mentha × piperita*) oil is a widely known source of natural flavorings characterized by a strong cooling sensation mainly provided by (−)-menthol, one of its most abundant compounds. In addition to this cooling effect, there are important components in peppermint oil that are key to the quality of its unique flavor characteristics such as the presence of (−)-menthone, methyl acetate, 1,8-cineole, (−)-limonene, and germacrene-D, as the most abundant chemosensates. Black Mitcham (BM) (*Mentha × piperita*), the leading peppermint cultivar in the United States for high-quality oil, is a sterile clone that was discovered in the 1,800 s (24) and has not been significantly improved since. Genomically, BM is a polyploid sterile plant (2n = 6× = 72) that had arisen from two hybridization and polyploidization events: an initial cross between diploid relatives *Mentha longifolia* and *Mentha suaveolens* generated allotetraploid spearmint (*Mentha spicata*). A second cross between spearmint and autooctoploid progenitor *Mentha aquatica* (2n = 8× = 96) generated peppermint (25). Recent genome assembly efforts have helped to characterize the diploid progenitors *M. longifolia* (26, 27) and *M. suaveolens* (28, 29), and produced molecular markers and genetic maps (26, 29, 30). A fragmented assembly of the BM genome was recently developed for comparative analysis with the diploid progenitors (31).

We present the development and characterization of a γ-irradiated population of peppermint cultivar BM. After developing a more contiguous genome assembly of BM, we report on indel size, type, and density. Using whole-genome sequencing dosage analysis, we showed that virtually all mutations are chimeric, affecting only specific layers as opposed to the whole plant body. Testing a subset of the mutant clones confirmed diversity in oil composition. Two independent individuals with drastically low (−)-menthol production were used for validation and found to both carry a deletion of the same allele of the (−)-menthone:(−)-menthol reductase enzyme. Taken together, our results provide a powerful resource for the study of layer-derived mutations analysis, as well as for mint functional genomics. Additionally, these results highlight the preponderance and phenotypic impact of layer-specific mutations in long-lived individuals. Our results are consistent with previous findings that periclinal chimeras dominate the world of somatic variants and that the dynamics and turn-over of layer-specific genomes within a long-lived clone are likely critical to their evolution and fitness. They also highlight the importance of developing methods for detecting and characterizing periclinal chimeras, as well as the potential impact of layer-specific engineering for plant improvement.

## Results

**Fig. 1.**
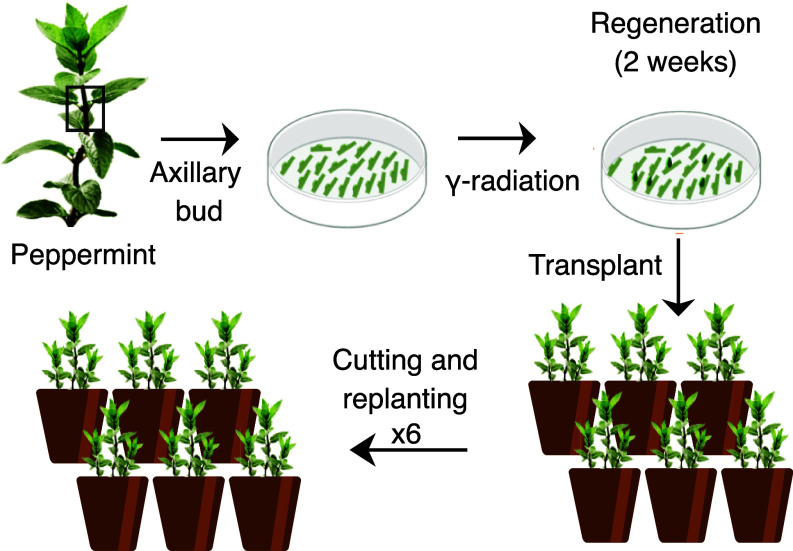
Peppermint population development. Axillary peppermint buds from cultivar BM (*Mentha × piperita*) were γ-irradiated by a ^137^Cs source using a 45 Gy dose. Plants recovered on agar for 2 wk before being moved to soil. Before field testing, plants were clonally propagated six times using single shoot tips as starting tissue, to reduce the frequency of chimeric plants (*Materials and Methods*).

To produce a peppermint mutant population, a total of 550 axillary buds from elite peppermint cultivar Black Mitcham (BM, *Mentha × piperita* L.) were γ-irradiated by a ^137^Cs source to create genetic diversity. After six rounds of shoot tip cuttings and replanting, aimed at minimizing mericlinal (sectorial) chimeric mutations, we obtained a total of 261 independent lines that were sequenced for genomic characterization of the induced mutations (Fig. 1).

### Most γ-Mutants Bear Several Large-Scale Indels.

To characterize the induced mutations in this population, we developed a genome assembly for the elite variety BM, the same accession used to produce the mutant population. BM is an allohexaploid (2n = 6× = 72), carrying genomic contributions from three different parental species: diploid *M. longifolia* (LL), diploid *M. suaveolens* (MM), and octoploid *M. aquatica* (AAAAAAAA) (32). Mint clones are typically heterozygous, and, therefore, we expected close to six haplotypes for each chromosome, of varying parental origins. Based on previous reports, the predicted haploid genome size of BM ranges between estimates of 1.37 Gb from ISSR markers (33), to 2.05 Gb using k-mer distribution (31).

A combination of PacBio HiFi reads and Hi-C reads were used to produce an assembly that contains 102 scaffolds > 5 Mb and for a total size of 1.783 Gb. This assembly includes between 6 and 11 contigs per chromosome, representing different haplotypes (homologs or homeologs) of either full chromosome length, or partial chromosome length (*SI Appendix*, Figs. S1 and S2 and Dataset S1). We named the contigs sequentially but also assigned them to specific chromosomes and included the chromosome information in the contig names. For example, the longest contig corresponding to chromosome 1 is labeled contig1_Chrom1A and spans the entire length of chromosome 1 (*SI Appendix*, Fig. S2). Within a chromosome type, contigs are labeled in decreasing order of size, with contigs labeled “A” being the largest of their group. For the remainder of this report, we will refer to contigs by their chromosomal assignment only (Chr01_A, etc.). More details about the assembled genome characteristics and its annotation can be found in the Methods section, and in Dataset S1. The total length of the selected scaffolds fall within the estimated genome size of BM (31, 33); however it does not contain the expected six copies of each chromosome. This could be due to the fact that some of the haplotypes, possibly those originating from autooctoploid *M. aquatica*, are not sufficiently differentiated to be assembled separately. It is also likely that highly repeated regions, such as centromeres, pericentromeres, and ribosomal DNA clusters are not fully included in the assembly because they are more challenging to assemble. Conversely, for some regions, more than the expected six haplotypes were assembled. This could potentially be due to aneuploidy or chimerism in BM, as can be expected from such a sterile and long-lived clone (24). This high contiguity draft assembly with separate haplotypes, while likely incomplete, is adequate for the purpose of our experiments.

**Fig. 2.**
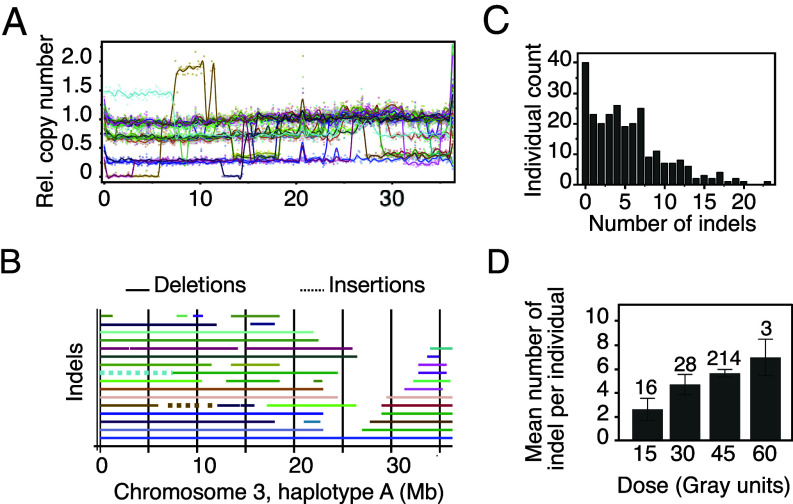
Genomic characterization of the γ mutant population. (*A*) Density and size of indels found on a single haplotype, using chromosome 3, haplotype A (Chr03_A) as an example. Normalized coverage curves for all individuals carrying at least one indel on this haplotype are shown. Values dropping below 1 represent deletions while insertions correspond to values higher than 1. (*B*) Representation of the physical location of all indels detected on Chr03_A. Deletions are depicted with solid lines while insertions are represented with dotted lines. (*C*) Distribution of the number of indel per individual in the whole population. (*D*) Increase in the number of indel per individual based on irradiation dose, error bars correspond to SEM (Table 1).

Armed with this assembly, we performed whole genome DNA sequencing of each of the 261 γ-mutants and searched for indels by normalizing read count coverage to the control nontreated BM. Using this approach, indels manifest as a series of chromosomal bins with contiguous reduced (deletion) or increased (insertion) copy number values, compared to BM (Fig. 2*A*). We detected on average 5.38 large-scale indels per individual, for a total of 1,406 indels (Dataset S2). Indels were divided into deletions (96.3%) and insertions (3.7%). They collectively covered each scaffold well, with an increasing number of indels closer to the chromosome ends, as expected from a collection of deletions that do not include the centromeres (Fig. 2 *A* and *B*). Overall, each annotated transcript was covered by multiple indels. Terminal arm deletions were predominant, and only a few chromosomal regions remained uncovered by indels. We speculate that these sparsely covered regions correspond to centromeres (Fig. 2*B*). Most indels covered partial chromosomes but, in a few cases, the entire chromosome was missing (aneuploidy, see Fig. 2 *A* and *B* and *SI Appendix*, Fig. S3). Not surprisingly, the shorter chromosomal contigs (Chr03_G, Chr05_I, and Chr09_H) were covered by the fewest indels (Supplementary file 2, *SI Appendix*, Figs. S3 and S4). Only 40 individuals (~15%) did not carry any indels, while some carried more than 20 indels (Fig. 2*C*). Examining the series of the plants used in the pilot experiment for the radiation dose–response curve, we noted that indel number increased with higher radiation doses (Table 1 and Fig. 2*D*) suggesting a direct effect of radiation dose on indel frequency.

**Table 1.** Lesion summary by dose

		Deletions			Insertions			All indels	
	N samples	N per sample	Total N	Mean size (Mb)	N per sample	Total N	Mean size (Mb)	N per sample	Total N
15 Gy	16	2.62	42	6.54	0	0	0.00	2.62	42
30 Gy	28	4.57	128	6.29	0.14	4	0.16	4.71	132
45 Gy	214	5.46	1,168	6.42	0.20	43	0.58	5.66	1,211
60 Gy	3	5.33	16	7.25	1.67	5	2.97	7.00	21

Mutation counts, sizes, and per-sample rates across γ radiation doses.

To assess whether the chromosomal breaks that resulted in the indels occurred randomly or if they clustered around potential hotspots, we documented the distribution of indel borders across the genome by recording the number of borders found in 1 Mb bins. Of 1,783 bins present in our genome assembly, only 675 (37.9%) did not harbor any indel border while the remaining bins harbored up to 17 borders per bin, but most bins harbored a single border (*SI Appendix*, Fig. S5). This distribution does not highlight any chromosomal region as potential hotpots of chromosomal breakage. Next, we assessed sequence context. Each border could only be defined to the nearest 100 kb bin because the mutants were sequenced at low coverage. We could not detect any significant difference in the sequence context (genic vs. nongenic) between the 100 kb bins that harbor indel borders compared to those that do not. It should be noted, though, that our assembly probably lacks some of the heterochromatic regions (centromeres, pericentromeres, and ribosomal clusters), reducing the power of our analysis.

### Most Mutants are Periclinal Chimeras.

**Fig. 3.**
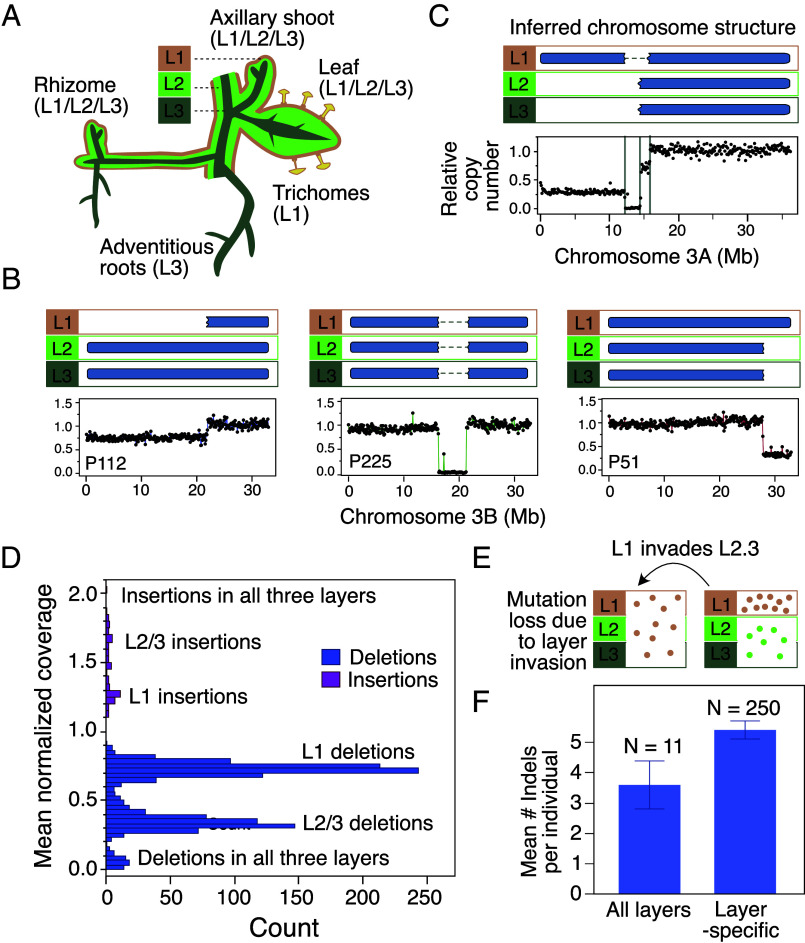
Layer-specificity of the indels. (*A*) Layered organization of plant architecture and layer-specific origin of different tissue types. (*B*) Different layer-specific deletion scenarios and dosage plot examples for each type. (*C*) Overlapping layer-specific deletions can result in full deletions. In this example, the relative dosage plot can be interpreted as follows: the top of Chr03_A is deleted in the epidermis (L1). In the same individual, a different deletion is found in the L2/3. The chromosome locations of the two deletions overlap, creating a ~2 Mb region for which all copies are deleted, resulting in complete loss of that chromosomal region in all layers. (*D*) Distribution of normalized coverage values found for all indels in the population. Values below 1 represent deletions while insertions correspond to values higher than 1. Deletions can be further subcategorized in L1 deletions (normalized coverage values ~0.7), L2/3 deletions (normalized coverage values around 0.3), or full deletions (dosage of 0 indicating loss in all three layers). Similarly, insertions can be categorized as L1, L2/3, or full. Deletions are depicted in blue and insertions in pink. (*E*) Model of the transitions between layer-specific mutations and fully mutated individuals through layer invasion. (*F*) In 11/261 clones, all indels exhibit a dosage close to 0 (deletions) or 2 (insertions), suggesting that these mutant clones are not chimeric. In those individuals, the mean number of indels is lower than in the chimeric individuals, consistent with the loss of the indels in one of the two layers upon homogenization. Error bars correspond to SEM.

Given that our assembly of the BM genome includes separate haplotypes, we expect that deletion of a specific region will result in a copy number of 0 and insertions result in a dosage of 2, compared to the control BM, which is expected to carry one copy of the corresponding region. Using this normalization, we found that most of the indels detected in the mutant population fell in specific intermediate dosage categories instead (Figs. 2*A* and 3), suggesting chimerism within the mutant individuals for those indels. The chimeric deletions fell into two main types: those with a relative copy number ~0.3 and those with a relative copy number ~0.7 (Fig. 3*D*). The fact that the ratio of mutant to wild-type (WT) copies is consistent between individuals irrespective of the leaf tissue sampled suggests a relatively stable mixture of cells that carry the indel and cells that do not. This pattern is not expected from sectorial or mericlinal chimeras, in which the percentage of mutated cells would vary significantly depending on the leaf sampled. It is, however, consistent with what we would expect in a periclinal chimera, where the relative percentage of L1 to L2/3 cells within a leaf remains similar across leaves and across samples. We observed a similar situation when mapping reads to haplotypic scaffolds in autotetraploid potato, in which we documented approximately 20% to 80% frequencies of L1 and L23 cells in leaves, respectively (2). The difference in relative percentages might reflect species-specific differences in layer abundance in leaves.

Based on these results, we hypothesize that most indels are chimeric deletions or insertions specific to epidermal layers (Fig. 3*A*). For example, loss of a region in the inner layers (L2- and L3-derived) produced a value of ~0.3 because the L1-derived cells have retained that region (Fig. 3*B*). Similarly, deletions in the outermost epidermal (L1) layer only produced a value of ~0.7 because the L2 and L3-derived cells have retained that region and account for ~70% of the cells (Fig. 3*B*). Insertions fall into two categories which can be interpreted similarly although they were much less frequent. Overall, L1-specific indels were more common than L23-specific indels (1.65× and 2.3× for deletions and insertions, respectively, Table 2).

**Table 2.** γ-indels summary by layer

Layer	Type	N	% of genome covered by at least one indel	% of transcripts covered by at least one indel	Mean size of indel (Mb)	Mean size, std error
All three	Deletion	46	17.59	19.70	7.24	0.92
All three	Insertion	5	0.88	0.95	3.13	2.78
L1	Deletion	769	88.88	92.20	7.71	0.22
L1	Insertion	23	5.64	5.30	4.11	1.23
L23	Deletion	467	76.90	82.60	7.28	0.29
L23	Insertion	10	2.48	2.50	3.75	1.97
All indels			96.26	98.43		

Indel counts, sizes, and percentage of genes and genome space covered by each indel type.

Dosage values close to zero correspond to deletions in all three layers and were rare (Fig. 3*D*). They either originated from overlapping layer-specific indels (Fig. 3*C*), or homogeneous individuals (Fig. 3*F*). In our population of 261 mutant individuals, we only found 11 individuals for which indels were consistently homogenous (full deletions with a dosage of ~0 or full insertion with a dosage of ~2, Fig. 3*F*). The 11 individuals that exhibited nonchimeric indels carried on average fewer indels than the rest of the population (Fig. 3*F*), as would be expected if the indels from one of the two layers had been lost after layer invasion (Fig. 3*F* and Table 2).

**Fig. 4.**
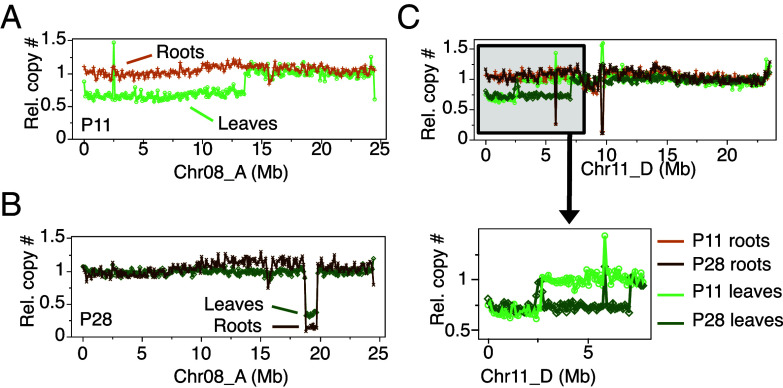
Layer-specificity of the indels detected in the genomes of P11 and P28 individuals. Roots are derived from pure L3 while leaves contain a mixture of cells derived from all three layers. When comparing the dosage curves in leaves and roots of the same individuals, we can infer the layer-of-origin of the indels. Examples are shown for deletions detected on Chr08_A (*A* and *B*) and Chr11_D (*C*). Data for all scaffolds exhibiting variation are presented in *SI Appendix*, Fig. S8. Copy number values are presented in Table 3. (*A*) In P11, a deletion with dosage values around 0.7 in leaves is not visible in roots (dosage close to 1), suggesting that it is only present in the epidermis. (*B*) In P28, a deletion with dosage values around 0.3 in leaves is fully deleted in roots (dosage close to 0), suggesting that it is only present in the tissues derived from the L2/3. (*C*) Mutant individuals share a deletion on Chr11_D. In both cases, the deletion is undetectable in roots, suggesting that it is restricted to the epidermis.

To confirm the hypothesis that most indels are layer-specific, we further characterized the indels present in the two low (−)-menthol mutant plants identified in our field screenings (P11 and P28, see below). Scanning both individuals for indels, we found 9 and 3 indels encompassing a total of 36.3 and 18.5 Mb in P11 and P28, respectively (Table 3). Both individuals carried chimeric indels with 7 out of 9 indels with normalized values above 0.6 coverage in P11 and 2 out of 3 in P28. To confirm the layer–specificity of the indels, we produced whole genome DNA sequencing of root tissue of P11, P28, and BM. Mint roots are adventitious and originate from the stem L3 during propagation. Therefore, roots consist exclusively of L3-derived cells while leaves contain a mixture of L1, L2, and L3-derived cells (Fig. 3*A*). If indels are layer-specific, we therefore expect that their dosage in the roots will be different from what it is in the leaves. Indeed, we found that the deletions that were present in leaf tissue with dosage values ~0.7 were absent in root tissue, and deletions with dosage values ~0.3 in leaf tissue were now fully represented in the roots. Two deletions were used to showcase this phenomenon in Fig. 4 (*SI Appendix*, Fig. S6 and Table 3 for the complete list of indels in P11 and P28). Both root tissues displayed an irregular dosage plot on Chr09_C (*SI Appendix*, Fig. S6) that was absent in the leaf samples. We cannot explain the origins of this pattern at this time, but it is not consistent with a regular indel (no clear edges). One plausible explanation is that it corresponds to a repeat-rich region for which copy number assessment is more challenging.

**Table 3.** Indels detected in low-menthol individuals P11 and P28

P#	Dose (Gray)	Chrom. haplotype	Start	End	Indel size (Mb)	Indel type	Mean Norm. Copy # in leaves	Mean Norm. Copy # in roots	Inferred mutated layer
P011	45	Chr03_B	31500001	32857933	1.36	Deletion	0.70	1.08	L1
P011	45	Chr03_E	17100001	18400000	1.30	Deletion	0.38	0.09	L2/3
P011	45	Chr04_A	33500001	34843660	1.34	Deletion	0.66	0.97	L1
P011	45	Chr04_K	1	7000001	7.00	Deletion	0.68	1.00	L1
P011	45	Chr05_F	6600001	7700000	1.10	Deletion	0.37	0.09	L2/3
P011	45	Chr07_B	900001	3200000	2.30	Deletion	0.80	0.99	L1
P011	45	Chr08_A	1	13500000	13.50	Deletion	0.68	1.04	L1
P011	45	Chr11_D	1	2500000	2.50	Deletion	0.73	1.03	L1
P011	45	Chr12_F	1	5900000	5.90	Deletion	0.70	1.09	L1
P028	45	Chr02_A	26700001	27400000	0.70	Deletion	0.73	1.01	L1
P028	45	Chr06_C	1	10200000	10.20	Deletion	0.71	0.97	L1
P028	45	Chr08_A	18800001	19800000	1.00	Deletion	0.35	0.14	L2/3
P028	45	Chr11_D	1	7000000	7.00	Deletion	0.74	1.06	L1

### Single Allele, Layer-Specific Indels can Affect Plant Function.

Next, we wondered if layer-specific modification of one haplotype in a hexaploid background was sufficient to affect plant function and be reflected in phenotypic variation. Field trials were conducted to test the performance of a subset of mutant lines preselected by a quick sensory greenhouse test for aromas that possessed unique characteristics. A total of 78 of the 261 mutant lines were observed over the course of 3 y (different lines were observed in different years). Each year, a control genotype (BM, C1) was included, and five traits were observed: height (cm), bloom percentage (%), ground cover percentage (%), dry biomass (ton/ha), and oil composition (measured by GC-FID, *SI Appendix*, Fig. S7 and Dataset S3). Significant variation compared to the BM control was observed for all traits measured. For example, clones P3, P16, and P29 presented earlier blooming, while P19, P30, and P52 produced shorter plants. P64 was the only individual with a statistically different biomass production compared to the control (+30%, *P* > 0.0113).

**Fig. 5.**
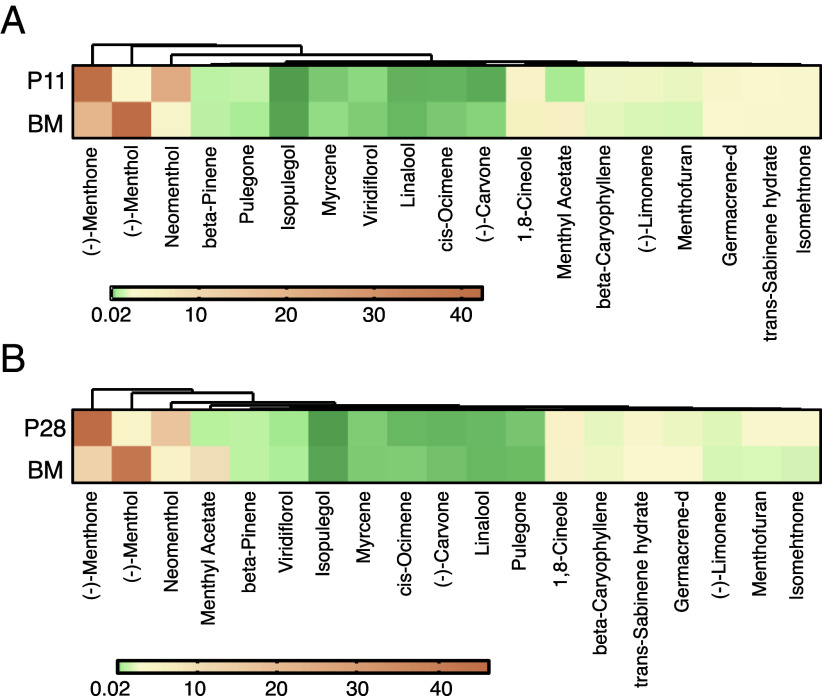
Two of the γ mutant individuals produce oils with altered profiles. Oil profiles identified when testing under field conditions. Colors represent mean abundance percentage values of three biological replications. For each mutant line, the corresponding control BM profile is shown as well. (*A*) Results from the 2019 field trial. (*B*) Results from the 2020 field trial (See *SI Appendix*, Fig. S3 for the complete set of lines tested in all years).

After the first field season (2019), one line stood out as exhibiting a unique oil composition profile when clustering individuals based on compound abundances (*SI Appendix*, Figs. S7*A* and S8 and Table S1). P11 presented reduced levels of (−)-menthol and menthyl-acetate, but increased levels of (−)-menthone and neomenthol when compared with the control BM (Fig. 5*A*). Specifically, (−)-menthol levels were reduced from 42 to 2.6% (12-fold, *P* > 6.00E-05), menthyl-acetate abundance was reduced from 4.5 to 0.54% (8.3-fold, *P* > 0.00158) while neomenthol and (−)-menthone levels increased 6.5-fold (from 3.47 to 22.5%, *P* > 0.000) and twofold (from 19.7 to 41.8%, *P* > 0.00028), respectively. Other less abundant compounds also increased slightly such as (−)-limonene and menthofuran while cis-ocimene, and isopulegol levels were reduced (*SI Appendix*, Table S1 and Dataset S1). In the following field season, using the same approach we identified another line, named P28, with oil characteristics similar to P11. P28 also presented reduced levels of (−)-menthol (from 42 to 4%, 10-fold, *P* > 0.0024) and menthyl-acetate (from 9.5 to 0.89%, 10.7-fold, *P* > 0.00238), as well as increased levels of (−)-menthone (from 12.8 to 46.5%, 3.64-fold, *P* > 0.00029), neomenthol (from 4.68 to 17%, 3.63-fold, *P* > 0.0021), and isomenthone (from 1.74 to 3.1%, 1.78-fold, *P* > 0.000) when compared with the control BM (Fig. 1*B* and *SI Appendix*, Figs. S7*B* and S8 and Table S2). We tested an additional set of lines in year three but none of the most abundant compounds were affected as drastically in those lines (*SI Appendix*, Fig. S8). Overall, the levels of (−)-menthol were inversely correlated with (−)-menthone and (+)-neomenthol (*SI Appendix*, Fig. S9).

### A Monoallelic Layer-Specific Deletion Is Sufficient to Drastically Alter Secondary Metabolite Levels.

**Fig. 6.**
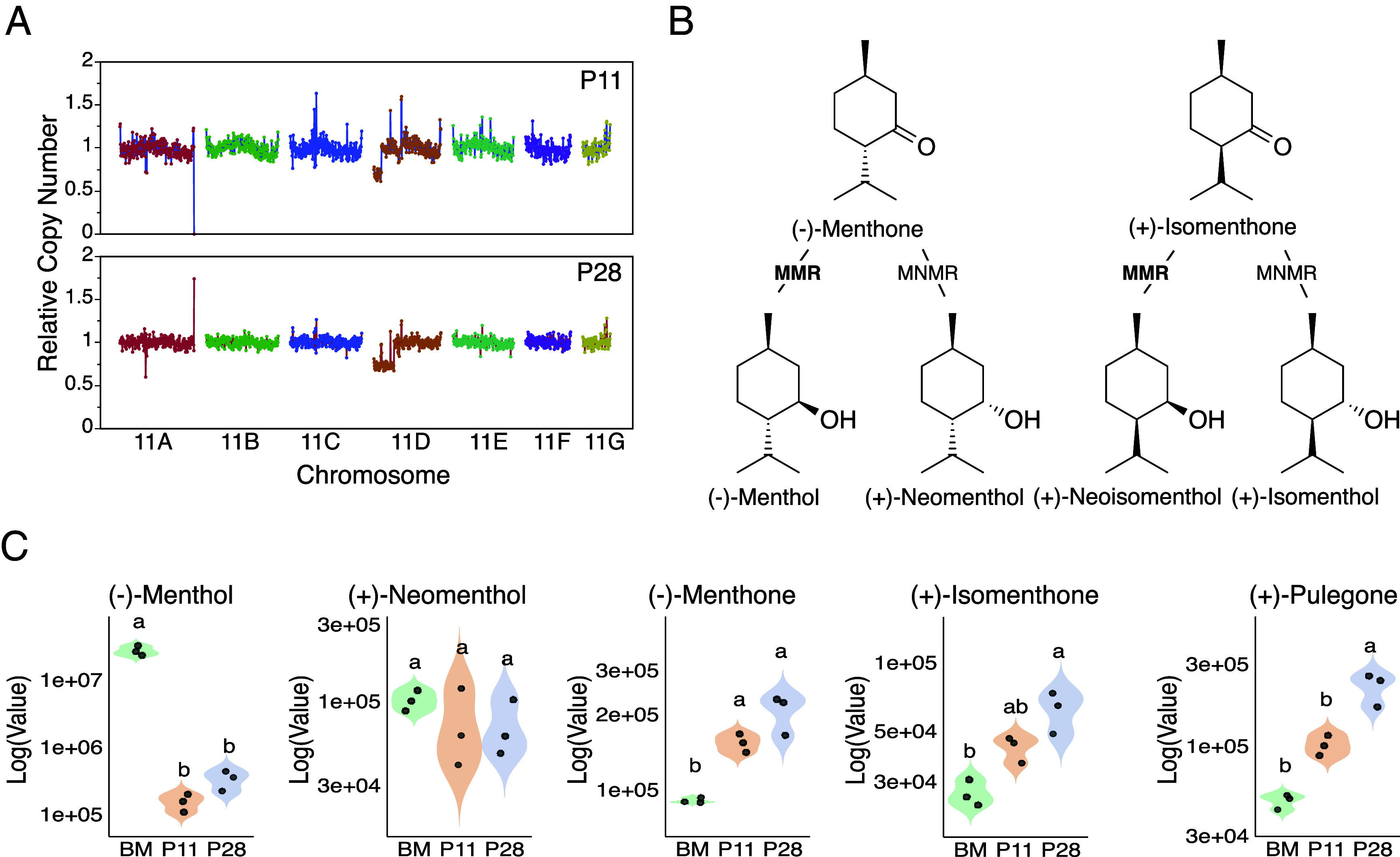
Effect of deletions of regions of chromosome 11 on oil profiles. (*A*) Chromosome 11 indel dosage plots for the P11 and P28 mutant individuals. Colored curves represent the normalized coverage obtained for each haplotype, each expected to be present in one copy in BM. Values of 1 indicate the absence of indel. Lower values are interpreted as L1 (0.7), L2/3 (0.3), or full (0) cell layer deletions. There is a single chromosomal region that exhibits a deletion in both mutants, located on Chr11_D (Fig. 5). (*B*) Role of the MMR and (−)-menthone:(+)-neomenthol reductase in the monoterpene biosynthesis pathway in *Mentha spp.* according to (35). (*C*) Quantification of compounds in leaves of BM, P11, and P28 individuals grown under controlled conditions, using UHPLC. BM: Black Mitcham control. Different letters indicate significantly different means (Tukey, *P* < 0.05). MMR, (−)-menthone:(−)-menthol reductase; MNMR, (−)-menthone:(+)-neomenthol reductase.

P11 and P28 both produced oils with low (−)-menthol levels (<10 to 12 fold compared to the control). To identify the gene(s) responsible for this phenotype, we first searched for regions deleted in both individuals. We found a unique haplotypic region that was deleted in both mutants, corresponding to Chr11_D (Fig. 4 and *SI Appendix*, Fig. S6). In P11, a deletion spanned the distal region between 0 to 2.5 Mb while, in P28, the first 7 Mb were deleted. Both deletions exhibit dosage values consistent with deletions in the L1-derived layer only. Among the 295 genes annotated within that region was BM19v5_g428725 (2,131,790 to 2,133,606 bp), which is predicted to be a (−)-menthone:(−)-menthol reductase gene (MMR) per our annotation. MMRs convert (−)-menthone to 95% (−)-menthol and 5% (+)-neomenthol (34), making it a strong candidate to explain the reduction in (−)-menthol and increase in (−)-menthone and (+)-neomenthol in these two mutant plants. MMR genes were also found in five other haplotypes of chromosome 11 (Dataset S1) but none of these other regions carried any indels in individuals P11 or P28 (Fig. 6*A*), confirming that only haplotype D was deleted in these mutants.

**Fig. 7.**
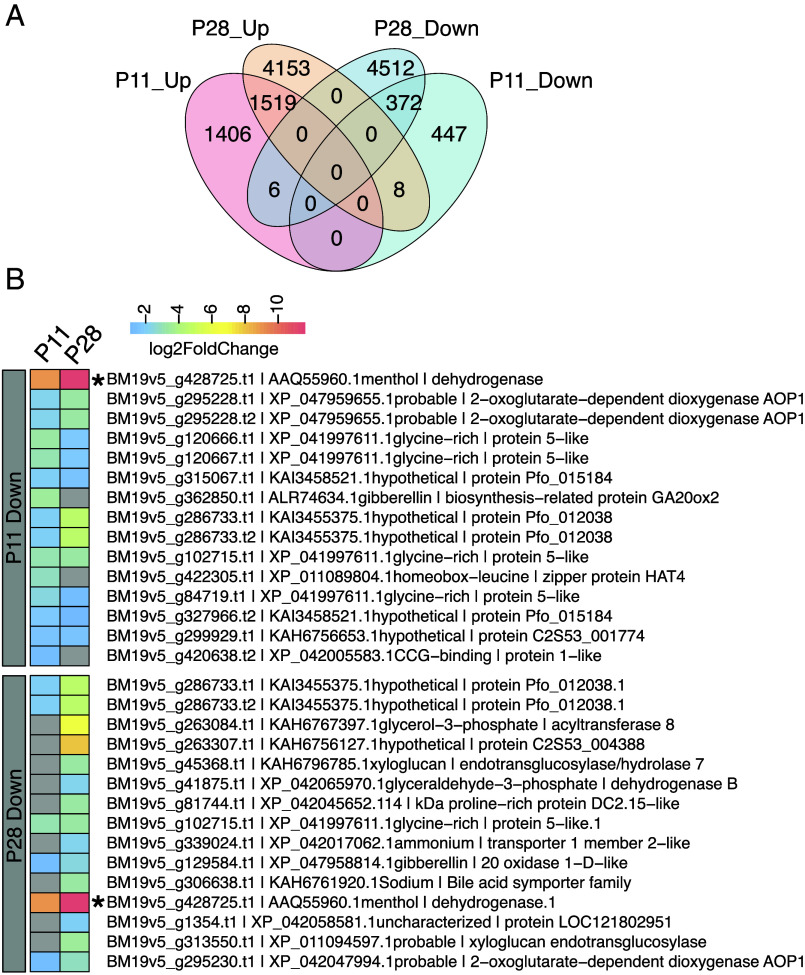
Differentially expressed genes in plants carrying indels in Chr11_D. (*A*) Number of genes differentially regulated in each mutant plant and corresponding intersections. (*B*) Top 15 downregulated genes in each mutant according to their *P*adj values. Colors indicate log2foldchange values (gray means no value). Gene BM19v5_g428725.t1 (marked with an asterisk) corresponds to the (−)-menthone:(−)-menthol reductase (MMR) mapped in the Chr11_D deletion.

To investigate the exact role of the loss of this MMR allele in the altered oil composition phenotype, we next grew P11 and P28 under controlled conditions to investigate differential gene expression associated with this phenotype. Leaf tissue was collected when approximately 10% of stems were flowering, which is similar to our field trial but also when plants would be harvested for commercial production. UHPLC-MS/MS analysis on leaves determined that P11 and P28 both exhibited reduced levels of (−)-menthol, as well as significantly increased levels of (−)-menthone compared to the control (Fig. 6*C*), confirming our observations under field conditions (Fig. 5). Differential Expression (DE) analysis was performed comparing expression in mature leaves in each mutant to the control BM. In P11, 3,758 genes were categorized as differentially regulated while, in P28, almost three times more genes with differential expression were detected (10,570). Of those, 1,905 genes were found to be differentially regulated in both mutants: 1,519 upregulated in both mutants, 372 downregulated in both mutants, and 14 genes differentially regulated in opposite directions (Fig. 7*A* and *SI Appendix*, Figs. S10–S12 for GO annotation of the DE genes). When analyzing the top downregulated gene in both mutants, we found that the MMR allele located on Chr. 11D (BM19v5_g428725, Fig. 7*B*) was downregulated in a similar fashion in both plants (~10-fold reduction).

We retrieved all predicted MMR alleles and confirmed that the BM19v5_g428725 allele (Chr11_D) was the only one to be differentially regulated in both plants (*SI Appendix*, Table S3). Gene BM19v5_g435105 (Chr11_E allele) was downregulated in P28 with a smaller change in expression (1.6-fold) in comparison with the Chr11_D allele. Notably, in the WT BM, the Chr11_D allele exhibits the highest expression levels. Alleles from haplotypes E, A, and C exhibit ~10-fold lower expression compared to the allele present on the D haplotype, and alleles from haplotypes G and B do not seem to be expressed in leaves at all (zero mapped reads, *SI Appendix*, Table S4). The coding sequence of the gene on Chr11_D also matches exactly the MMR cDNA from peppermint (AAQ55960.1) isolated from mature secretory cells of trichomes (34). The other MMR alleles were 82 to 92% diverged and formed distinct phylogenetic branches suggesting differentiation (*SI Appendix*, Fig. S13). Consistently, two other mutants (P30 and P55) carry a deletion of the G allele of MMR. Based on our previous indel analysis, the deletion in P30 is present in all layers and the deletion in P55 was present in the epidermal layer only. In P30 and P55 oils, the levels (−)-menthone, (−)-menthol and neomenthol were similar to those observed in BM, confirming MMR activity despite the loss of the G copy (*SI Appendix*, Fig. S7 and Dataset S3).

Last, we checked if other genes commonly deleted in both mutants could modify the oil profiles. One of the 295 genes deleted in both mutant individuals, BM19v5_g428729, corresponds to a predicted (−)-menthone:(+)-neomenthol reductase (MNMR) (*SI Appendix*, Fig. S12). This enzyme converts (−)-menthone into (+)-neomenthol and (+)-isomenthone to (+)-isomenthol (Fig. 6*B*). Looking at expression read counts of the MNMR alleles in the BM control, we found that the homeolog located on the G haplotype is the dominant allele, with expression an order of magnitude higher than the other alleles (BM19v5_g428729, *SI Appendix*, Table S4). Taken together, these results suggest that this enzyme is unlikely to be responsible for the observed phenotype.

## Discussion

Our characterization of a γ-irradiated population of peppermint led to two major findings, with substantial implications for plant breeding, genetics, and plant evolution.

### All Somatic Mutations are Layer-Specific, At Least at First.

Our first finding validates Sophia Satina’s original discovery: most persistent somatic mutations originate in a single cell and spread within that layer through anticlinal divisions, resulting in layer-specificity (1, 2, 36). Occasionally, periclinal divisions can result in layer invasion and either loss or homogenization of the mutation to the whole organism.

In our population of γ mutants, we found that most indels are layer-specific and that homogeneous indels were rare (Fig. 3), confirming that layer invasions only arise with relatively low frequency. We also showed that these indels persisted in a layer-specific arrangement from mutagenesis until approximately 1 y later, when we sampled tissue for sequencing. During that time, we propagated the tissue multiple times, indicating that they are stable through generations of plant propagation. At the point of sampling, we only found 11/261 mutants in which the mutations were homogeneous (not layer-specific). We hypothesize that these individuals started out with layer-specific mutations as well, but that, at a later time, one meristematic cell from one layer invaded the other layers and populated subsequent growth, resulting in homogeneously mutant individuals (Fig. 3*E*). Alternatively, it is possible that layer invasion occurred right after mutagenesis, either because of meristematic restructuring associated with the stress of the mutagenesis itself, or because mutations in one of the cell layers were so severe that the cells of that layer were unable to proliferate and were replaced by the other layer.

We further observed that indels were more common in the L1 layer compared to the L2/3 layer. This is consistent with recent findings in potato (2) and apricot (1), and with the hypothesis that the L2 meristematic layer might have evolved to be more protected against new mutations because of its role in producing the germline. The fact that this bias is also visible in our population mutated with a penetrating agent suggests that the bias does not originate from higher exposure of the epidermis because of its physical location.

### Homeolog Specialization Provides a Path Toward Chemotype Variation.

The second finding is that this method is efficient for a forward genetics screen in a sterile clonal and polyploid crop that cannot be selfed and subjected to traditional mendelian analysis (37–42). Applying a dose of 45 Gy, we produced up to 39 indels per chromosome and deleted each annotated transcript on average six times. With this system, we identified variation in oil profiles at relatively high frequencies (*SI Appendix*, Fig. S8). Focusing on variation in oil profiles, we found two mutants with strongly reduced levels of (−)-menthol and increased levels of (−)-menthone and neomenthol (*SI Appendix*, Fig. S7 and Dataset S3). The large phenotypic effect was surprising in the context of MMR redundancy: all six alleles and homeologs encode full-length proteins but the Chr11_D allele is responsible for (−)-menthol production in peppermint.

Many of the genes involved in the mint oil biosynthetic pathway are physically linked, forming clusters of related genes with different enzymatic functions (27), similar to the biosynthetic gene clusters found in many other organisms (43, 44), including plants (45) and Lamiaceae (46, 47), and involved in the production of specific secondary metabolites. The MMR gene identified in this study is tightly linked to the phylogenetically closely related (−)-menthone:(+)-neomenthol reductase (MNMR) gene, which encodes another well-characterized enzyme of menthol metabolism (Fig. 6*B* and Dataset S1). The MMR and MNMR enzymes compete for the same two substrates, (−)-menthone and (+)-isomenthone for the production of (−)-menthol and (+)-neoisomenthol by MMR, or the production of (−)-neomenthol and (+)-isomenthol by MNMR (Fig. 6*B*). The D allele of both genes is deleted in mutants P11 and P28, but the observed oil composition is consistent with the loss of MMR activity but not with the loss of MNMR activity. Correspondingly, the G allele of MNMR exhibits the highest expression in BM, suggesting a potential dominant role of the G haplotype in (−)-neomenthol synthesis.

In summary, the enzymes controlling two competing pathways appear to be encoded in the same gene cluster, but in repulsion (on different haplotypes). Furthermore, a single major allele controls a key trait in a complex polyploid. This finding implies easy genetic separation of distinct chemotypes in glandular trichomes, increasing population variability of oil composition and the connected capacity to resist biotic and abiotic stresses (48). Accordingly, the existence of a single dominant functional allele facilitates manipulations to confer relevant traits with practical applications. Consistent with this, bias in dominant homeolog gene expression has been described in polyploid wheat, with approximately 30% of wheat genes displaying nonbalanced expression from its three subgenomes (49, 50), as well as in other allohexaploids such as cotton (51).

### Layer-Specific Variants Have Evolutionary Consequences and Practical Applications.

Historically, humans selected L1 chimeras for their commercial value. For example, the *meunier* trait of Pinot grapevine is valuable in champagne production and has been maintained for hundreds of years (52). Here, we show that induced deletions specific to a single layer and a single haplotype result in a significant change in oil composition. This suggests that an epidermal phenotype modifying, for example, defense compounds could significantly alter fitness of an individual (48). In long-lived clones, this modification could be propagated vegetatively for very long periods of time. Additionally, this modified L1 genome could eventually invade the L2 and achieve sexual transmission, especially in the presence of selective pressure for fit progeny. Recently, single-cell transcriptome analyses in apricot have also shown that layer-specific mutations are only detected in the transcriptome of the cells of the corresponding layers (1). These results confirm that layer-specific genotypes can be associated with specific transcriptomes, metabolomes, and, generally speaking, novel phenotypes.

The formation of layer-specific somatic mutations has important implications on breeding long-lived clonal crops. A trait encoded in the L1 will not be transmissible through gametes short of a rare layer invasion. A chimeric trait will only be present in tissue culture regenerants originating from a specific layer (2, 21). Knowledge of periclinal status will thus be important in breeding of certain species. Selection of periclinal mutants may also be useful. It could be advantageous by altering a specific layer while retaining the phenotypic characteristics of the other layers. In peppermint, for example, this could be applied to the specific engineering of oil profiles or production by modification of the L1 layer, as exemplified here. Similarly, specific modifications to the L3 layer could result in changes in root physiology that alter disease response without compromising oil metabolites. A recent study demonstrated that mint produces different oil compounds in different organs (53). Furthermore, the amount and type of oil produced changed upon inoculation with *Verticillium dahliae*, the soil-borne fungus responsible for Verticillium wilt, a disease associated with significant yield loss in mint production. The ability to separately engineer different layers could prove a powerful and flexible approach to improvement in crops with long-lived clones in general.

### Conclusions.

It has been almost 90 y since John Stadler initiated the first radiation experiments, using a portable X-ray equipment in an effort to increase crossing-over effects in maize, translating the approach from previous Drosophila studies (54). The approach presented here, coupling ionizing radiation with current sequencing technologies, and the implementation of transcriptomic studies, is perfectly suited to lay down the foundation for research hypotheses based on the understanding of layer-specific genetic regulation of plant phenotypes and their implications. Furthermore, detecting, characterizing, and potentially engineering layer-specific mutations could prove instrumental in the investigation of many aspects of plant biology associated with layer-specific developmental programs, such as stomatal function, trichome production, pigmentation, production, and release of defense compounds, or endoreduplication.

## Materials and Methods

### Population Development.

The plant material used in this study corresponds to the variety “BM” peppermint (*Mentha × piperita*, PI 557971, USDA NPCGRS, Corvallis, OR). We produced a dose–response curve using 40 axillary buds from the cultivar BM grown in MS media (no sucrose) by a ^137^Cs source at the Center for Health and the Environment (University of California, Davis, CA) (*SI Appendix*, Fig. S14*A*). The initial pilot study ranged from 40 to 200 Gy. The second test ranging from 0 to 80 Gy showed a survival response curve where 45 Gy presented a survival of 41% which was chosen as an appropriate dose to develop the population (*SI Appendix*, Fig. S14*B*).

Next, 550 axillary meristems were harvested from runners of well established greenhouse-grown plants, washed with sterile water twice and bleached with 10% bleach for 10 min under slight agitation. They were next rinsed three times with sterile water and placed in petri dishes with MS media (15 mm plates, *SI Appendix*, Fig. S14*C*). After treatment with a dose of 45 Gy at a rate of 553.33 Rad/min (Center for Health and the Environment, University of California, Davis, CA), plants were moved to food-grade clear plastic containers with the same media (*SI Appendix*, Fig. S14*D*) and grown for 2 wk before being moved to greenhouse conditions (*SI Appendix*, Fig. S14*E*). A total of 261 plants survived this treatment and moved to the next step, in which they were each propagated six times using tip cuttings in order to reduce the frequency of chimeric plants.

### Plant Phenotyping.

Field trials were conducted at the University of California Intermountain Research and Extension Center field station at Tulelake, CA (41°57′54.8″ N 121°28′14.5″ W), following previously published procedures (30). Briefly, 45 plants were transplanted from greenhouse grown cuttings in a plot of 10 × 12 ft (40 m2) area. A randomized complete block design was used with three replications per genotype. Each genotype was evaluated for traits measured to assess differences in growth, development, and oil quality. Statistical analysis was conducted using a fixed-effects linear model to estimate genotype effects, followed by Dunnett’s test for pairwise comparisons of each genotype against the control (BM, C1), with *P*-values adjusted to control for multiple testing. This approach allowed for the identification of genotypes significantly differing from the control in mean trait values, with significance thresholds set at *P < 0.05 and **P < 0.01. The analysis was implemented in R, using the dplyr and emmeans packages to compute genotype means, mean differences, SE, CI, and *P*-values, ensuring statistical inference.

For oil composition analysis of the plants used for transcriptome analysis, plants grown under controlled conditions were used as source of leaf tissue for ultra-high performance liquid chromatography tandem mass spectrometry analysis as previously described (55).

### Peppermint Genome Assembly and Annotation.

Young leaves from BM mature plants were collected and frozen for high molecular weight DNA extraction followed by PacBio HiFi library construction and sequencing at the UC Davis DNA Technologies Core, or nuclei extraction and Omni-C library construction (Dovetail Genomics) at UC Santa Cruz Paleogenomics laboratory, UCSC, followed by Illumina 150PE sequencing at the UC Davis DNA Technologies Core.

PacBio HiFi produced 16,355,327 reads with 8,187 bp mean length. To reduce memory requirements for the assembly, only reads over 10 kp length or high confidence consensus sequences (CCS reads with 10 passes or more) were retained. This represented ~85 percent of the reads, for a total of 13,835,311 reads at a mean length of 9,437 bps. These reads were combined with 1,073,209,059 PE Hi-C reads and assembled using the hifiasm assembly software in the joined hifi + Hi-C mode (56). All haplotypes were retained using the hifiasm -l 0 parameter to disable all purging of duplications. This parameter was used in conjunction with the --n-hap 6 parameter to indicate an expected number of six haplotypes. This resulted in 1,692 contigs representing 2.07 Gb of assembled space, with a mean length of 1.2 Mb, and N50 of 23.9 Mb. To create a limited chromosome scaffold set, only contigs larger than 5 MB were retained (N = 98).

To assign contigs to chromosomes, all >5 Mb contigs were mapped to a combined mint genomic reference consisting of the combination of the published genomes of *M. suaveolens* (28), *M. longifolia* [diploid, (27)] and *M. aquatica* [octoploid, (57)]. Repeated minimap2 mappings were used in conjunction with D-Genies visualization to determine clear contig to chromosome assignments and manually split chimeric contigs or remove fully chimeric/ repeat segment contigs. This resulted in 102 pseudochromosomes, with an overall N50 of 23.4 Mb and a total length of 1.78 Gbps. For each chromosome, between 7 and 10 contigs were retained and named according to decreasing order of size (A though G-J) for each chromosome. We expect that partial chromosome contigs could be further associated with each other to create additional full chromosome-size scaffolds but, without linkage information, we elected to keep them separate. For each of these 102 contigs, approximate homeologous regions on the *M. suaveolens* and *M. longifolia* genomes can be identified in *SI Appendix*, Fig. S1. The resulting BUSCO score to the eudicots_odb10 set was C: 97.9% (S:1.4%, D:96.5%), F:0.2%, M:1.9%, n:2,326. Scores for individual haplotypes can be found in Dataset S1.

Gene annotation was performed using BRAKER version 3.0.1 (58), using RNA_Seq data from 24 samples, representing a total of 1.05 billion reads, of which ~828 million mapped to the BM assembly. These 23 samples included four samples from mature leaf samples collected in field plots, 12 samples from mature leaves collected from flowering greenhouse-grown plants, as well as one sample each of mature leaves, buds, flowers, roots, runners, stems, and young leaves collected from greenhouse plants. The resulting unfiltered gene prediction set totaled 514,468 models representing a 527 Mb space. From this set, a filtered set was generated to reduce transposable elements and superfluous de novo genes. To this end, the gene models were put through omicsbox (Biobam Bioinformatics Solutions, S.L., Valencia, Spain) for annotation, searching for blast hits, GO terms, and PFAM annotation. The models that had any annotation data were then filtered by taking only models with 20 or more coverage from mapping 679 million PE RNA-Seq BM reads to the full gene set. The resulting filtered transcript set contained 188,435 models, which corresponds to an average of 188,435/6 = 31,405 transcript per genome.

In order to mine the newly assembled genome for the presence of mint biosynthetic genes specifically, we compiled a list of previously documented biosynthetic genes in peppermint and spearmint (http://langelabtools.wsu.edu/mgr/pathways) and used blast-2.16.0 to find homeologous genes in our reference genome, with the following criteria: blastn with an e-value cutoff of 1e-120. The list of genes can be found in Dataset S1.

### DNA Indel Characterization.

Leaf material was harvested from each γ-mutant and the control Black Mitcham peppermint (BM) were harvested, flash frozen, and used for DNA extraction (DNeasy Plant Mini Kit, Qiagen). For root DNA sequencing, mint cuttings were taken from a mature greenhouse plant and grown in a 50 mL tube of water. Cuttings each had 3 to 5 nodes, and all except the top node and meristem were stripped of their leaves. Plants were grown in a sunny windowsill and a water level was maintained to keep at least the lowest two nodes submerged. Roots regenerated from the nodes and lowest portion of the stem and were harvested after ~3 wk of growth. Rooted cuttings were rinsed under running DI water for ~1 min each. Roots were patted dry with a paper towel and sliced using a sterile razor blade. The entirety of the roots were collected, with ~1/2 cm left on the cutting stem. These harvested roots were immediately crushed in a clean liquid nitrogen-chilled mortar and pestle. Samples were ground for ~5 min with liquid nitrogen and stored at −80 °C until DNA extraction.

For all DNA samples, we checked DNA quality and quantification prior to moving forward with library preparation. All genomic sequencing libraries were prepared using the KAPA DNA HyperPrep as recommended by the manufacturer, except that all reactions were used at half volume. Library concentrations were measured using a QuBit 2.0 fluorometer. Samples were pooled and sequenced using either the Element Biosciences AVITI sequencing system or the Illumina NovaSeq available through the UC Davis DNA Technologies core.

Reads were demultiplexed and processed to trim N-bases in reads, check for adapter contamination, and maintain a 5-bp sliding window of read base phred score 20 or higher. The reads were then mapped using BWA mem version 0.7.17 (59) to create sam mapping files to the largest 102 scaffolds of the BM genome assembly. Next, for each individual, the pair-ended mapped reads of phred quality 40 or better were pooled into nonoverlapping 100-kb bins on the scaffolds, and bin read mean coverage was normalized to the mean coverage of a control BM library for the same bin using a custom script called bin-by-sam (https://github.com/ComaiLab/bin-by-sam).

A custom python script (https://github.com/ComaiLab/binning_cnv_detection) was used next on a per library, per scaffold, basis to identify indels. When five or more sequential bins were detected as exhibiting increased or decreased dosage compared to the control, the script would extend out the detected indel until it ended. The run parameters used were --pen 25, --min-size 5, --del-cut 0.80, --ins-cut 1.15, --gap-bins 30, --post-core-cut 0.85, --post-run-bins 10, --extend-win 10, --extend-frac 0.6. Finally, all indels identified were visualized and checked manually. When necessary, the boundary of the indel was adjusted by hand. The final list of indels found in the γ mutant population can be found in Dataset S2.

### Transcriptomic Analysis.

Plants were grown under a temperature of 22 °C/18 °C, long day conditions (18 h light) and a light intensity of ~260 μM m^−2^ s^−1^ in a growth chamber (PGR15, Conviron, Manitoba, Canada) at the UC Davis Controlled Environment Facility. Samples were collected when plants reached ~10% bloom (i.e., approximately 10% of the shoots produced flowers), the same stage used to extract oil in field experiments. Mature leaves were harvested for RNA extraction and flash-frozen in liquid nitrogen and stored at −20 °C. Four replicated biological samples were collected at the same time from P11, P28, and the BM control each, and processed in parallel. RNA was extracted using the RNEasy Plant Mini Kit (Qiagan), following the manufacturer’s recommendations. After quality control, RNA-Seq libraries were developed using the KAPA mRNA Hyper Prep., following the manufacturer’s recommendations. In-house 8-bp dual indexed adapters were used. Sequencing was carried out at the DNA Technologies and Expression Analysis Cores at the UC Davis Genome Center.

Raw sequenced reads were first processed for quality using trim_galore version 0.6.7 (60) to trim N-bases in reads, check for adapter contamination, and maintain a sliding window of read base phred score 20 or higher. These reads were then mapped to the genomic fast of the 102 superscaffolds of our assembly using STAR version 2.7.11b (61), and with a modified filtered gene set GTF for guidance. This modified filtered gene set contained all 188,435 models present in the main filtered gene set plus any gene model identified in the indel regions of mutants P11 and P28, bringing the total to 196,974 models. The mapped SAM files were then put through subread featureCounts version 1.6.3 (62) to count mapped transcripts for each library, in pair-ended mode using the parameters -O for overlapping models allowed and -Q 40 for a minimum mapping quality of 40. This count file was then processed using DESeq2 (63) to look for differentially expressed genes, with normalization performed for four replicates per condition, and conditions run pairwise looking for DE transcripts with an adjusted *P*-value below 0.05. To expand the mapping to cover specific MNMR genes of interest not originally included in the filtered transcript set, htseq-count (64) was used to count the transcript reads in the RNA-seq libraries. This was done using default parameters with modifications to use a minimum mapping quality of 40, and specify that the input was not strand-specific.

### Data Visualization.

To generate heatmaps for visualizing gene expression data, we utilized several R libraries for data processing and visualization. The tidyverse package, a collection of R packages for data manipulation and visualization, was employed for handling of data frames, particularly through dplyr for filtering and joining operations and stringr for string manipulation of gene IDs. The pheatmap package was used to create the heatmaps, and display log2foldchange values across samples. The package superheat was used to plot oil profiles data. Additionally, gridExtra and grid were used to manage graphical outputs. A Pearson correlation matrix was computed using the cor function to quantify pairwise relationships between samples based on their chemical profiles. Statistical significance of correlations was assessed with the cor.mtest function, generating *P*-values at a 95% confidence level. The results were visualized using a correlation plot (corrplot), with significance levels (0.001, 0.01, and 0.05) indicated by symbols, and the matrix was ordered by angular order of eigenvectors to enhance interpretability of clustering patterns.

## Supplementary Material

Appendix 01 (PDF)

Dataset S01 (XLSX)

Dataset S02 (XLSX)

Dataset S03 (XLSX)

## Data Availability

Sequencing data are available through NCBI SRA Bioproject PRJNA1374144 (65). This includes the following: BM PacBio HiFi and Omni-C reads, RNA-Seq reads from both P11/P28 mutants, genomic short-reads from leaves from all γ mutants and the BM unirradiated control, genomic short-reads from roots of the P11 and p28 mutants, BM Illumina control reads. The assembled genome of BM (102 contigs) and its annotation was deposited in Figshare (https://doi.org/10.6084/m9.figshare.28457522) (66). The RNA-Seq reads from different BM tissues used for genome annotation were previously deposited as part of NCBI Bioproject PRJNA1226583 (67).
