# Supplementary material for: Layer-specific genetic variation unlocks secondary metabolite diversity in long-lived clonal peppermint
Source: Proc Natl Acad Sci U S A. 2026 May 8;123(21):e2532794123. doi: 10.1073/pnas.2532794123 (PMC13214039; doi:10.1073/pnas.2532794123)
Supplement: Supplementary file 1 — Appendix 01 (PDF) [file pnas.2532794123.sapp.pdf]

Supplemental Materials for

Layer-specific genetic variation unlocks secondary metabolite diversity in long-lived clonal peppermint

Nestor Kippes[1], Meric C. Lieberman[1], Darrin Culp[2], Isabelle J. DeMarco[1], Helen T. Tsai[1,3], Kanae Masuda[1], Jordan Lopez[4], Robert G Wilson[2], Luca Comai[1,5*], and Isabelle M Henry[1*]

[1] Department of Plant Biology and Genome Center, University of California at Davis, California 95616, United States

[2] University of California Cooperative Extension, ANR, Intermountain Research, and Extension Center, Tulelake, CA, 96134, United States

[3] Current affiliation: State of California, Dept. Financial Protection and Innovation

[4] Ingredient Science, Mars Wrigley, 1132 W. Blackhawk St., Chicago, IL 60642, United States

[5] Chulalongkorn University, Bangkok Thailand

Corresponding author
Isabelle M Henry
UC Davis Genome Center
Tel: +1(970)-261-1975
Fax: 530-754-9658
imhenry@ucdavis.edu

Luca Comai
UC Davis Genome Center
Tel: +1(530)-752-8485
Fax: 530-754-9658
lcomai@ucdavis.edu

Compound	Black Mitcham	P11	fold-difference	*p*	Significance
**Neomenthol**	3.470	22.553	6.50	0	***
**(-)-Menthone**	19.683	41.820	2.12	0.00028	***
**Pulegone**	0.597	1.160	1.94	0.135	
**(-)-Limonene**	1.753	2.163	1.23	0.01902	*
**Menthofuran**	1.673	2.030	1.21	0.03509	*
**Viridiflorol**	0.330	0.390	1.18	0.16403	
**Isomenthone**	2.393	2.750	1.15	0.02186	*
**beta-Caryophyllene**	1.930	2.180	1.13	0.1451	
**beta-Pinene**	1.000	1.020	1.02	0.36959	
**trans-Sabinene-Hydrate**	2.720	2.637	-1.03	0.59226	
**Germacrene-d**	2.483	2.353	-1.06	0.10245	
**1,8-Cineole**	4.873	4.407	-1.11	0.03318	*
**Linalool**	0.190	0.153	-1.24	0.01478	*
**Myrcene**	0.433	0.313	-1.38	0.4542	
**cis-Ocimene**	0.310	0.170	-1.82	7.00E-05	***
**(-)-Carvone**	0.363	0.107	-3.39	0.12281	
**Isopulegol**	0.060	0.017	-3.53	0.00605	**
**Menthyl-acetate**	4.513	0.540	-8.36	0.00158	**
**(-)-Menthol**	42.303	2.657	-15.92	6.00E-05	***

Table S1. Oil characterization in P11 versus the Black Mitcham control under field conditions. *t-test* based on 3 biological replications for each sample for data obtained during the first field season. Significance levels are denoted as: *** ($p < 0.001$), ** ($p < 0.01$), * ($p < 0.05$), or blank ($p \geq 0.05$).

Compound	Black Mitcham	P28	fold-difference	*p*	Significance
beta-Bourbonene	0.477	1.903	3.99	0.00437	**
(-)-Menthone	12.800	46.557	3.64	0.00029	***
Neomenthol	4.680	17.003	3.63	0.0021	**
Isomenthone	1.743	3.107	1.78	0	***
Menthofuran	1.913	3.090	1.62	0.02099	*
para-Cymene	0.060	0.087	1.45	0.02482	*
Pulegone	0.167	0.230	1.38	0.13692	
Piperitone	0.497	0.620	1.25	0.18252	
Isovaleraldehyde	0.030	0.037	1.23	0.56657	
(-)-Limonene	1.840	1.980	1.08	0.20929	
Linalool	0.147	0.153	1.04	0.43819	
Myrcene	0.247	0.257	1.04	0.72865	
1,8-Cineole	4.433	4.537	1.02	0.72078	
beta-Pinene	1.043	1.060	1.02	0.6874	
alpha-Pinene	0.817	0.827	1.01	0.77165	
Sabinene	0.577	0.570	-1.01	0.76768	
X1-Octen-3-ol	0.083	0.080	-1.04	0.7769	
Terpinolene	0.067	0.063	-1.06	0.51852	
γ-Terpinene	0.160	0.150	-1.07	0.6835	
trans-Sabinene-Hydrate	2.913	2.703	-1.08	0.20541	
alpha-Terpinene	0.073	0.067	-1.09	0.6401	
alpha-Humulene	0.110	0.100	-1.10	NA	
cis-Sabinene-hydrate	0.067	0.060	-1.12	0.42265	
beta-Caryophyllene	2.477	2.200	-1.13	0.04485	*
beta-Farnesene	0.607	0.513	-1.18	0.01869	*
X3-Octanol	0.153	0.127	-1.20	0.03856	*
Viridiflorol	0.503	0.410	-1.23	0.30135	
cis-3-Hexyl-isovalerate	0.517	0.410	-1.26	0.44538	
Terpinene-4-ol	0.187	0.147	-1.27	0.30956	
Germacrene-d	3.047	2.387	-1.28	0.02588	*
(-)-Carvone	0.200	0.140	-1.43	0.02131	*
alpha-Terpineol	0.077	0.053	-1.45	0.00776	**
Isomenthol	0.393	0.240	-1.64	0.00012	***
cis-Ocimene	0.260	0.147	-1.77	3.00E-04	***
Isopulegol	0.057	0.017	-3.35	0.03267	*
Neoisomenthol	1.097	0.320	-3.43	0.00019	***
Isopulegone	0.507	0.143	-3.55	0.00796	**
(-)-Menthol	42.877	4.057	-10.57	0.00024	***
Menthyl-acetate	9.513	0.890	-10.69	0.00238	**

Table S2. Oil characterization in P28 versus the Black Mitcham control under field conditions. *t-test* based on 3 biological replications for each sample for data obtained during the first field season. Significance levels are denoted as: *** ($p < 0.001$), ** ($p < 0.01$), * ($p < 0.05$), or blank ($p \geq 0.05$).

Gene	Chr.	Class	log2FoldChange_P11	log2FoldChange_P28	pvalue_P11	pvalue_P28
**BM19v5_g407383.t1**	Chr11_A	Not Significant	NA	NA	NA	NA
**BM19v5_g414295.t1**	Chr11_B	Not Significant	NA	NA	NA	NA
**BM19v5_g421841.t1**	Chr11_C	Not Significant	NA	NA	NA	NA
**BM19v5_g428725.t1**	Chr11_D	Down_Down	-8.947	-11.622	1.83E-57	1.12E-28
**BM19v5_g435105.t1**	Chr11_E	P28_Down	NA	-1.608	NA	1.49E-08
**BM19v5_g444822.t1**	Chr11_G	Not Significant	NA	NA	NA	NA

Table S3. Differential expression of MMR alleles. The chromosome D allele (BM19v5_g428725.t1) is the only transcript differentially regulated in both mutant individuals.

Geneid	Annot.	Chr	BM	BM	BM	BM	P11	P11	P11	P11	P28	P28	P28	P28
**BM19v5_g428725.t1**	**MMR**	**Chr11_D**	565	399	237	931	1	2	0	5	0	0	1	0
**BM19v5_g435105.t1**	MMR	Chr11_E	67	39	27	94	64	79	85	56	36	58	17	20
**BM19v5_g407383.t1**	MMR	Chr11_A	79	50	14	83	259	460	21	24	233	95	48	114
**BM19v5_g421841.t1**	MMR	Chr11_C	22	17	5	43	60	57	7	7	152	53	21	52
**BM19v5_g444822.t1**	MMR	Chr11_G	1	0	0	0	2	3	0	1	1	3	1	2
**BM19v5_g414295.t1**	MMR	Chr11_B	0	0	0	0	0	0	0	0	0	0	0	0
**BM19v5_g407340.t1**	MNMR	Chr11_A	33	16	19	29	55	144	65	85	75	55	29	33
**BM19v5_g407388.t1**	MNMR	Chr11_A	0	0	1	2	4	2	0	2	0	0	1	1
**BM19v5_g435105.t1***	MNMR	Chr11_E	67	39	27	94	64	79	85	56	36	58	17	20
**BM19v5_g444827.t1**	**MNMR**	**Chr11_G**	546	389	636	951	1492	1946	955	909	633	701	297	471
**BM19v5_g428729.t1**	MNMR	Chr11_D	5	6	4	7	10	38	5	6	0	5	0	5
**BM19v5_g428729.t2***	MNMR	Chr11_D	0	0	0	0	0	0	0	0	0	0	0	0

Table S4. **Read counts of MMR and MNMR enzyme copies detected in Chromosome 11.** BM: Black Mitcham control. P11 and P28 (-)-menthol deficient mutants. Columns represent biological replications. Note that, in the control, the D haplotype copy of the MMR is the dominant allele while, for the MNMR enzyme, the homeolog located on haplotype G is responsible for most of the gene expression observed. MMR, (-)-menthone:(-)-menthol reductase; MNMR, (-)-menthone:(+)-neomenthol reductase.

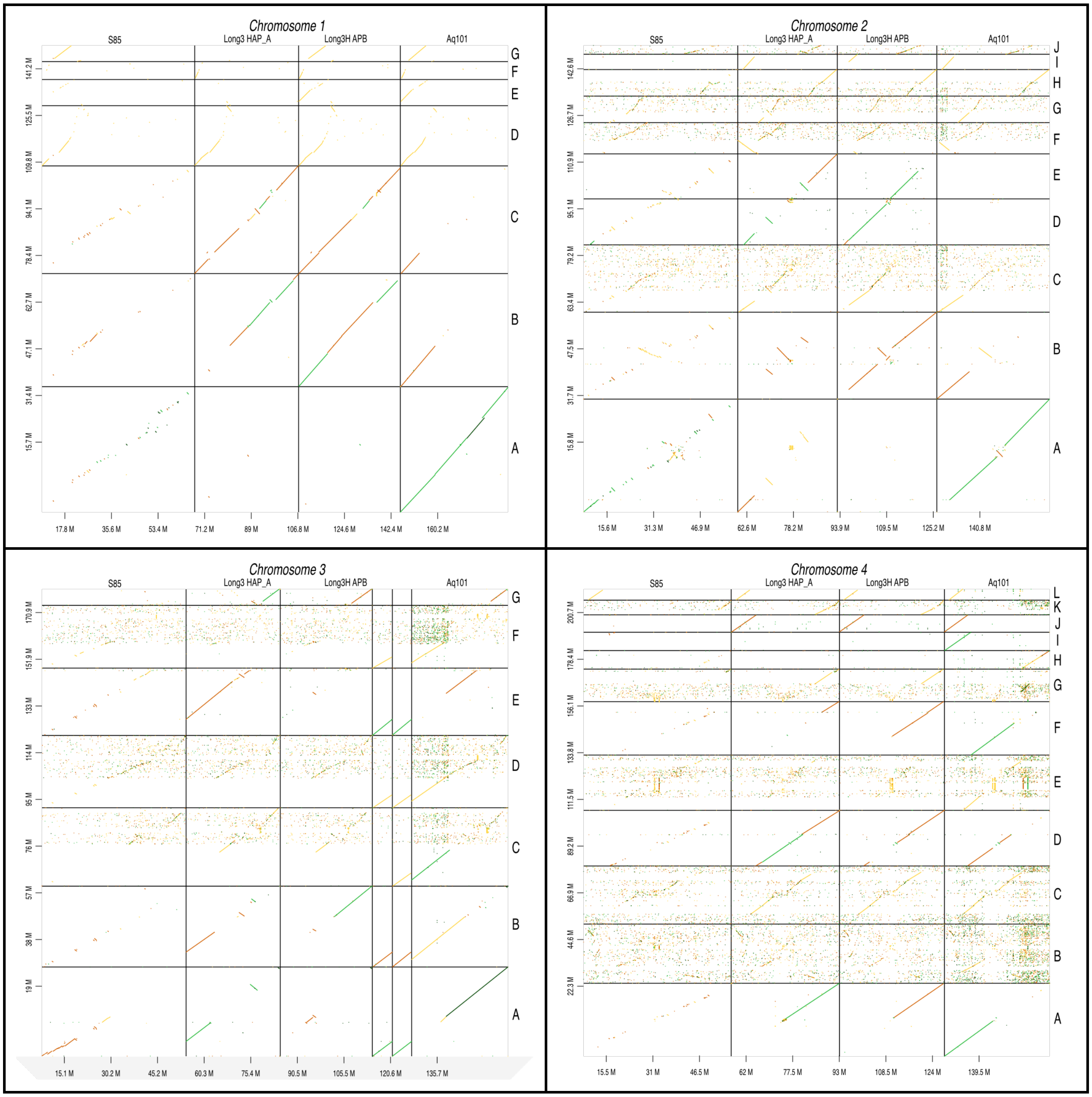

Figure S1. Chromosome assignment of the 102 contigs in the BM assembly. For each contig in the final assembly, the mini-map 2 alignment to the assembled genomes of *M. suaveolens* (consensus assembly), *M. longifolia* (2 haplotypes for each chromosome) and *M. aquatica* (consensus assembly) are shown against the various haplotypes assembled from BM for that corresponding chromosome (letters on the right).

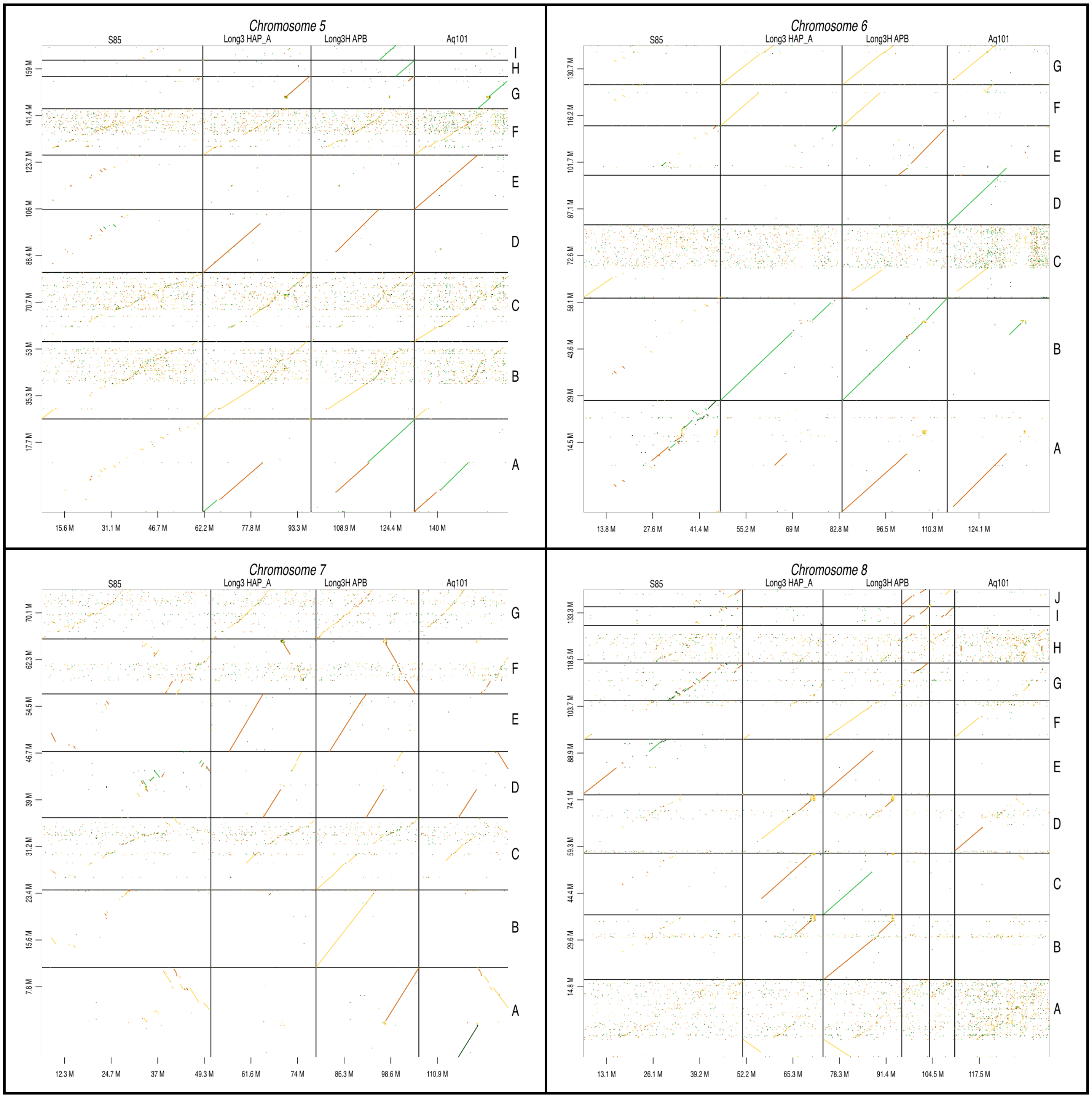

Figure S1 continued. Chromosome assignment of the 102 contigs in the BM assembly. For each contig in the final assembly, the mini-map 2 alignment to the assembled genomes of *M. suaveolens* (consensus assembly), *M. longifolia* (2 haplotypes for each chromosome) and *M. aquatica* (consensus assembly) are shown against the various haplotypes assembled from BM for that corresponding chromosome (letters on the right).

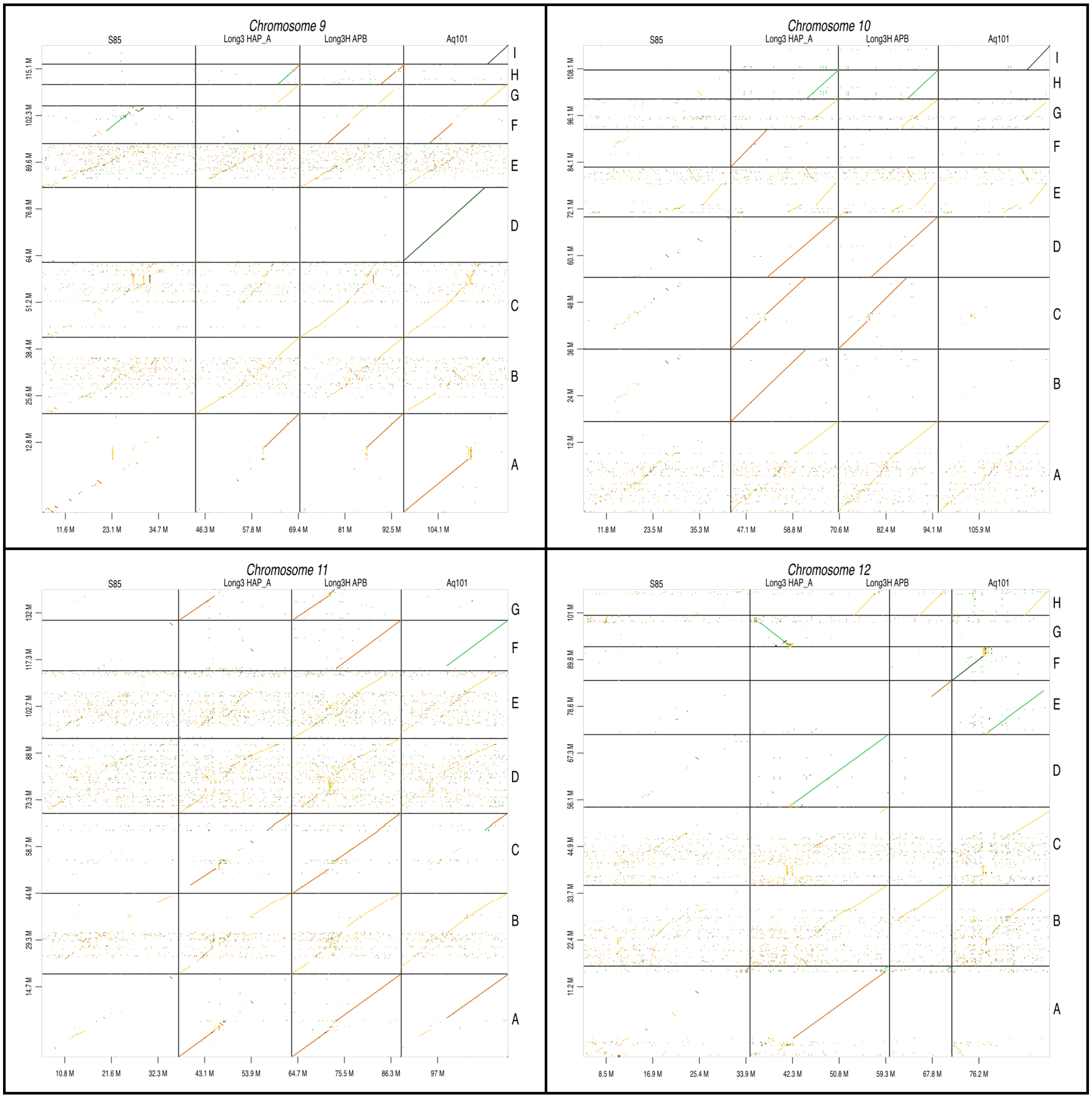

Figure S1 continued. Chromosome assignment of the 102 contigs in the BM assembly. For each contig in the final assembly, the mini-map 2 alignment to the assembled genomes of *M. suaveolens* (consensus assembly), *M. longifolia* (2 haplotypes for each chromosome) and *M. aquatica* (consensus assembly) are shown against the various haplotypes assembled from BM for that corresponding chromosome (letters on the right).

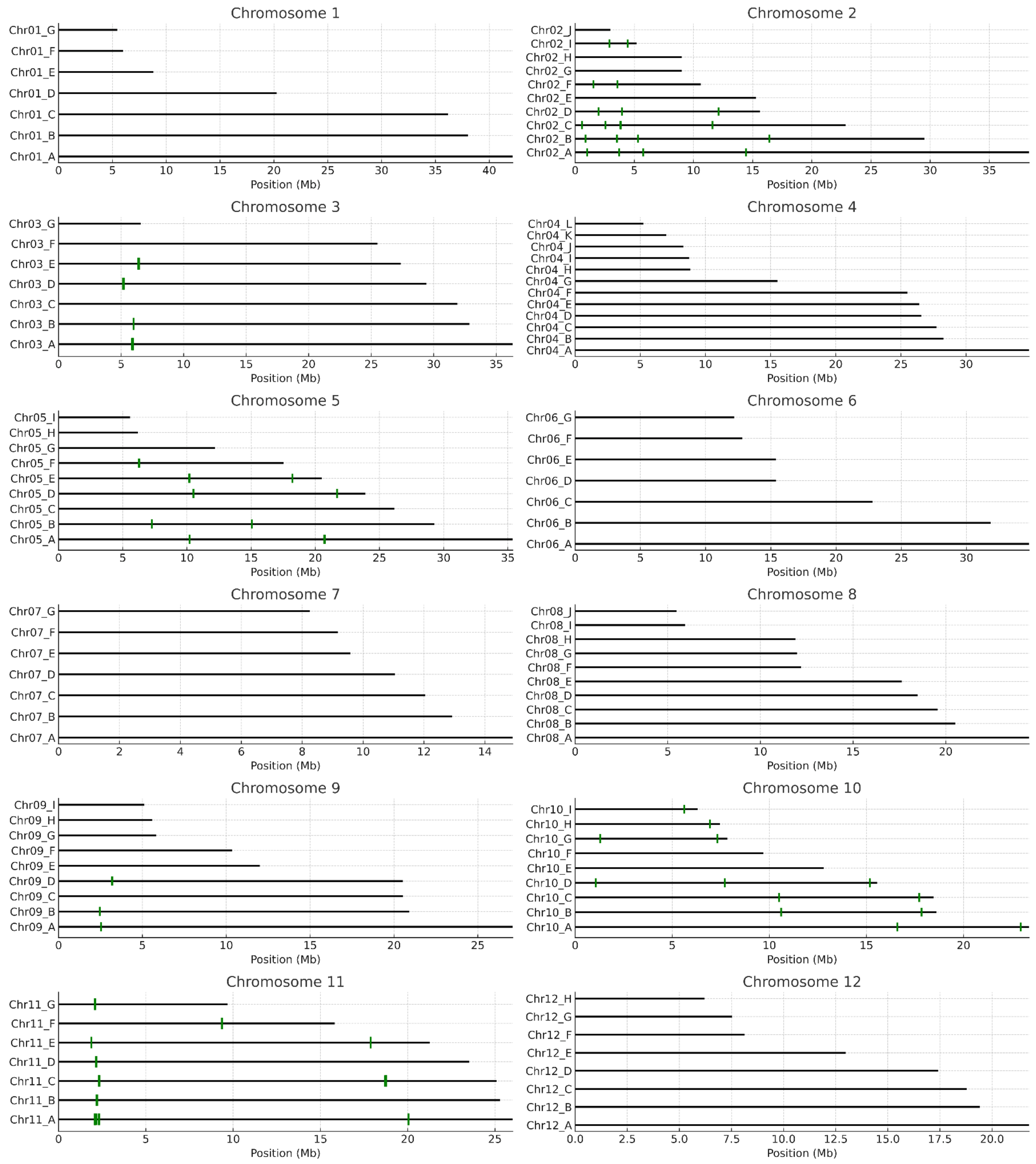

Figure S2. Oil biosynthetic genes identified in the BM assembly. For each chromosome type, the contigs (different haplotypes) associated with that chromosome are shown in decreasing order of size. Genes associated with mint oil biosynthesis (Dataset S1) are indicated by green tick marks.

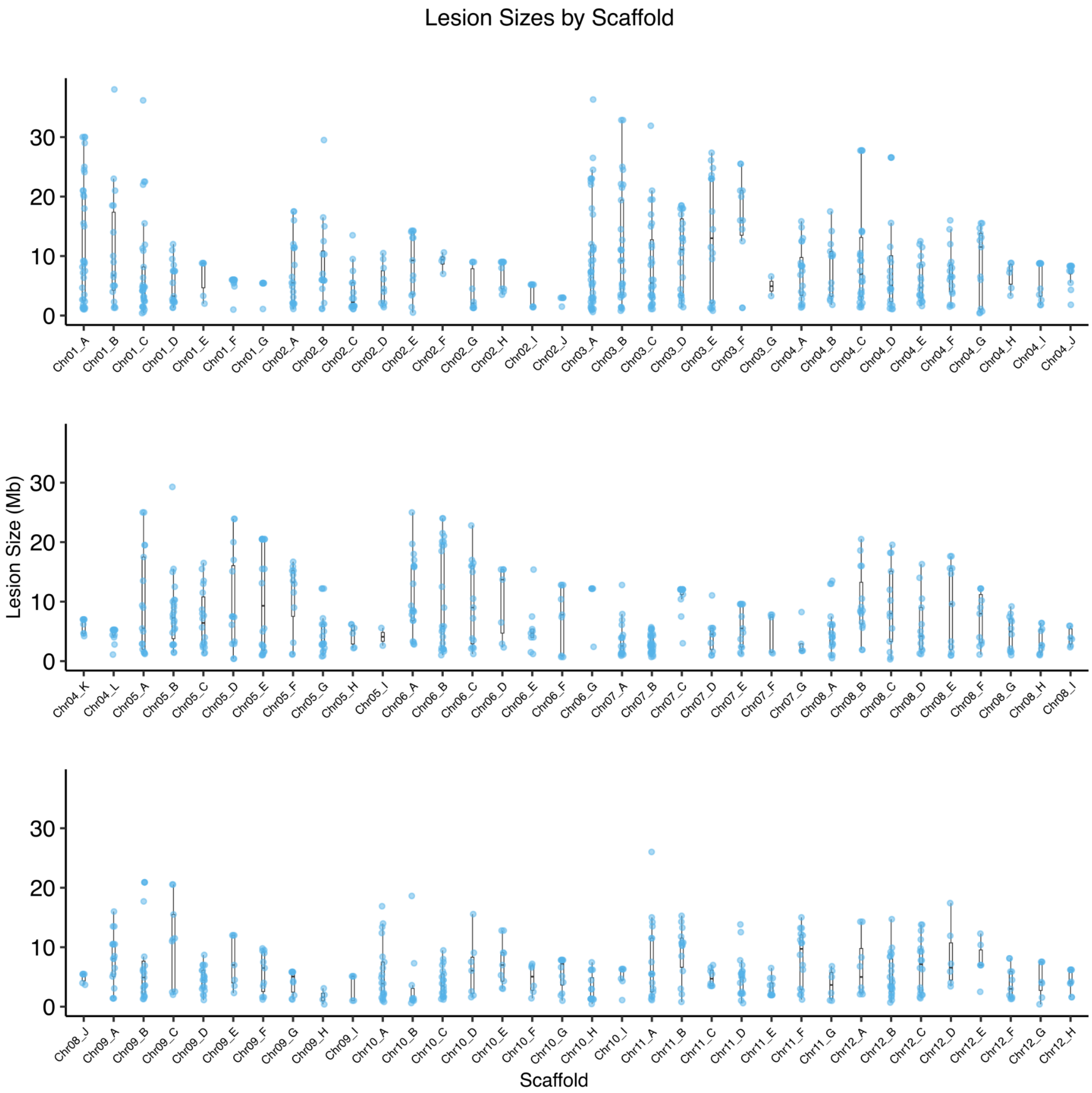

Figure S3. **Distribution of indel sizes for each scaffold.** Dots represent individual indel size. For each scaffold, the mean and standard deviations are indicated by dot plots in black.

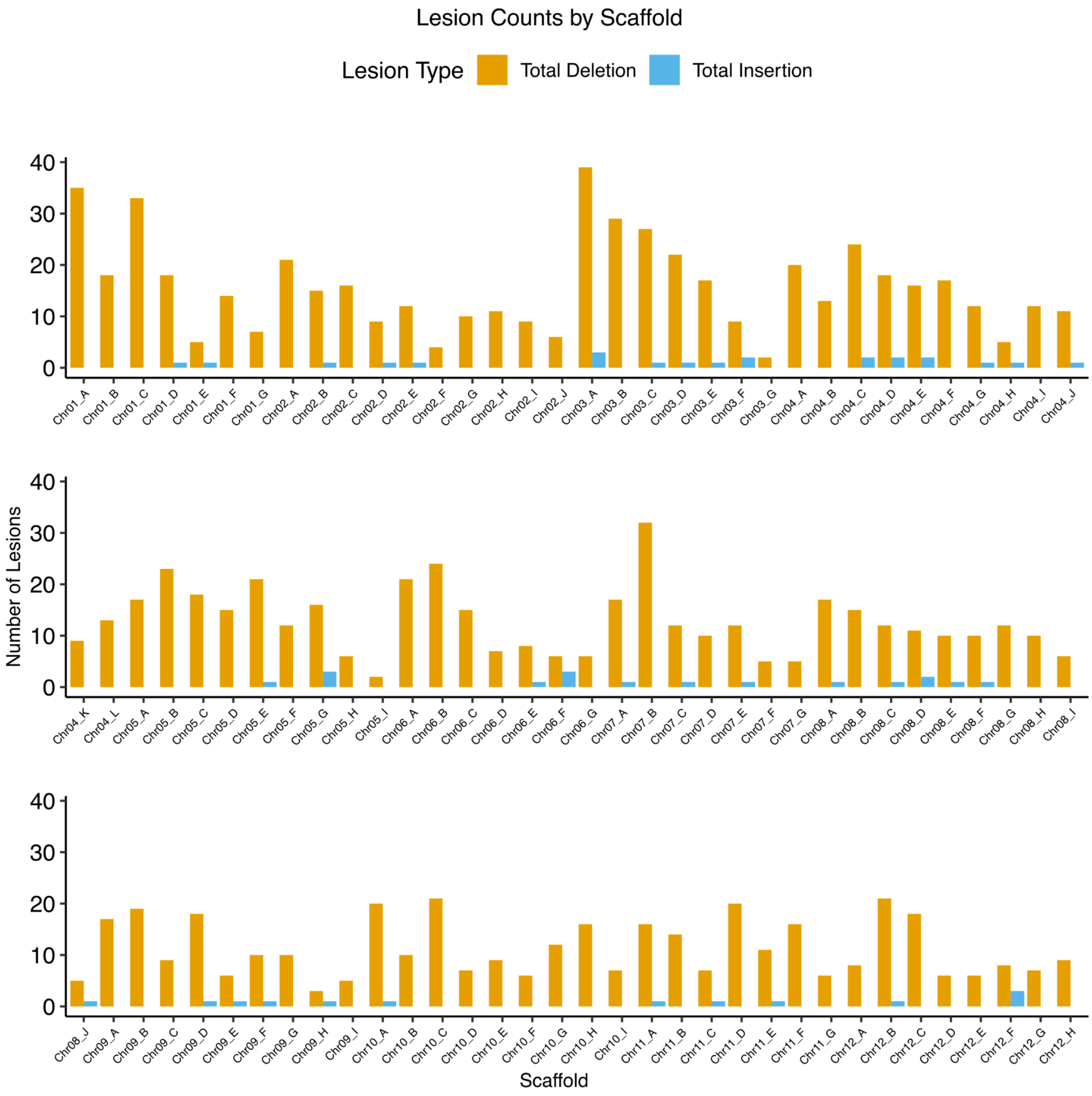

Figure S4. **Indel counts by scaffold.** Bar plots represent the total number of lesions found in the population for the corresponding scaffold.

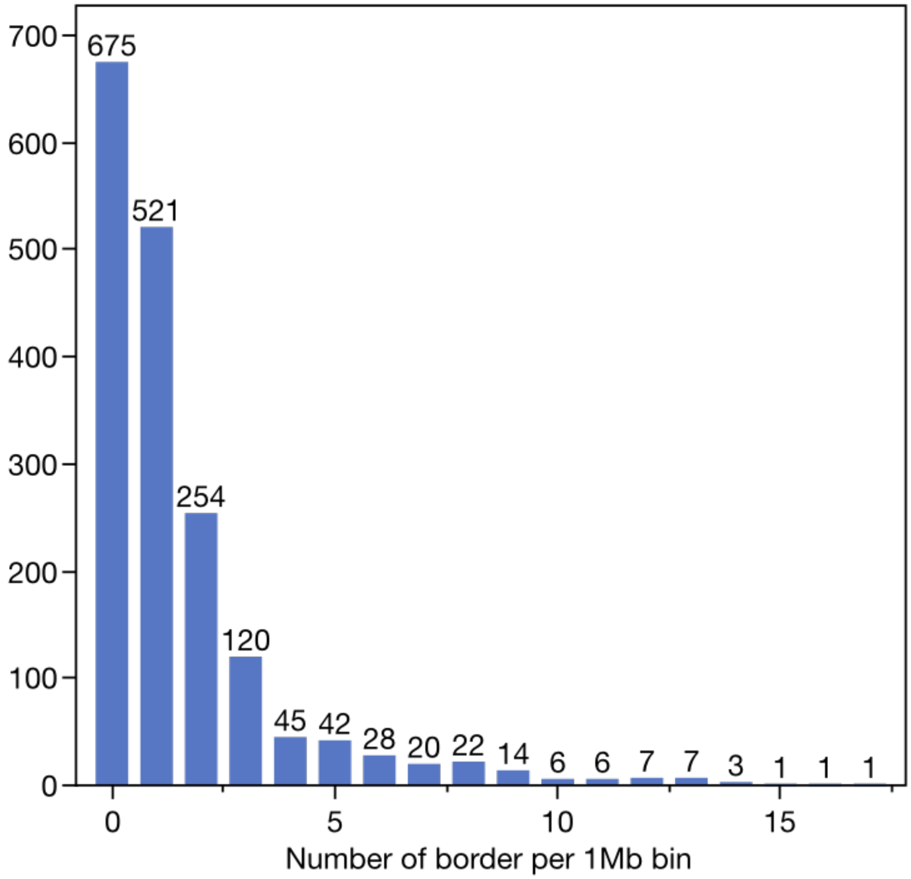

Figure S5. Genomic location of indel borders. For each indel, the location of the right and left border was recorded in increments of 1Mb bins. For each bin, the number of indel borders falling into that bin was counted. The draft genome includes a total of 1,783 1Mb bins. Of those, 675 (37.9%) do not harbor any indel border while the remaining bins harbor at least one and up to 17 borders. The distribution of border number per bin does not highlight the presence of hotspot for chromosome breakage.

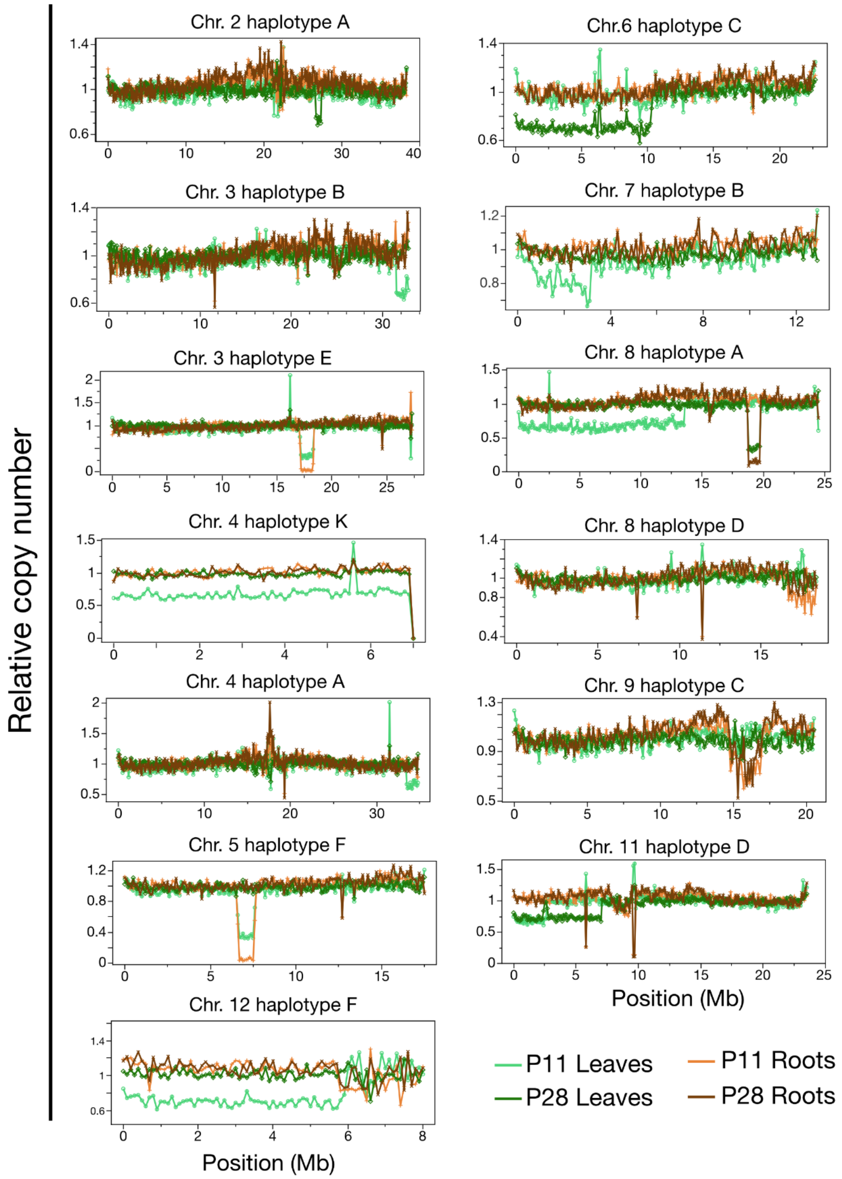

Figure S6. Layer-specificity of the indels detected in the genomes of P11 and P28 individuals. Roots are derived from pure L3 while leaves contain a mixture of cells derived from all three layers. When comparing the dosage curves in leaves and roots of the same individuals, we can infer the layer-of-origin of the indels. For P11 and P28, in all cases, deletions with dosage values around 0.7 in leaves are not visible in roots (dosage close to 1), suggesting that they are only present in the epidermis. Similarly, deletions with dosage values around 0.3 in leaves are fully deleted in roots (dosage close to 0), suggesting that they are only present in the tissues derived from the L2/3. Scaffolds for which variation was detected in leaves (green) or root (brown) tissues are shown. Values close to 1 indicate the absence of indels. Exact copy number values are presented in Table 3.

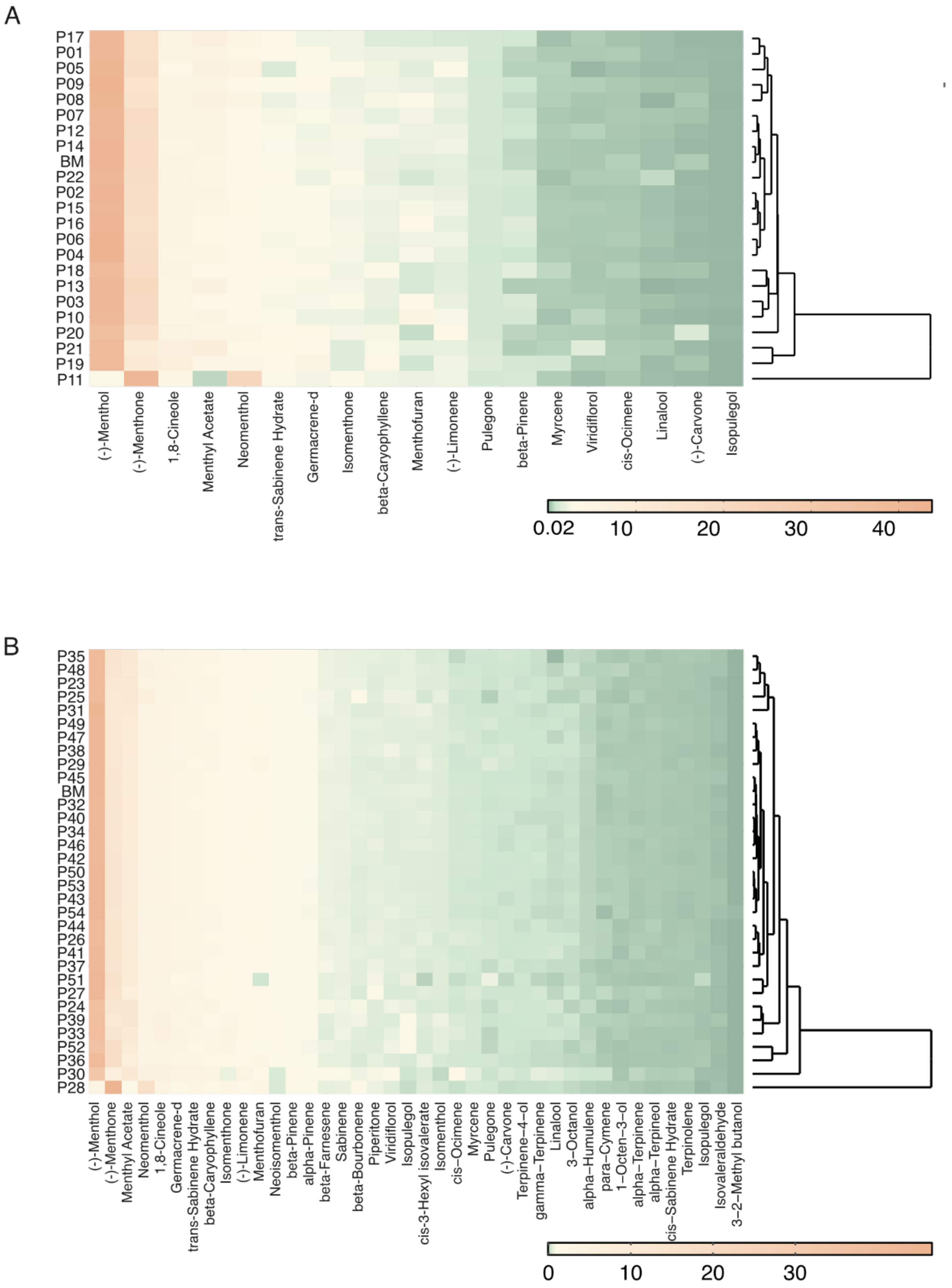
A
P17
P01
P05
P09
P08
P07
P12
P14
BM
P22
P02
P15
P16
P06
P04
P18
P13
P03
P10
P20
P21
P19
P11
(-)-Menthol
(-)-Menthone
1,8-Cineole
Menthyl Acetate
Neomenthol
trans-Sabinene Hydrate
Germacrene-d
Isomenthone
beta-Caryophyllene
Menthofuran
(-)-Limonene
Pulegone
beta-Pinene
Myrcene
Viridiflorol
cis-Ocimene
Linalool
(-)-Carvone
Isopulegol
0.02
10
20
30
40
B
P35
P48
P23
P25
P31
P49
P47
P38
P29
P45
BM
P32
P40
P34
P46
P42
P50
P53
P43
P54
P44
P26
P41
P37
P51
P27
P24
P39
P33
P52
P36
P30
P28
(-)-Menthol
(-)-Menthone
Menthyl Acetate
Neomenthol
1,8-Cineole
Germacrene-d
trans-Sabinene Hydrate
beta-Caryophyllene
Isomenthone
(-)-Limonene
Menthofuran
Neoisomenthol
beta-Pinene
alpha-Pinene
beta-Farnesene
Sabinene
beta-Bourbonene
Piperitone
Viridiflorol
Isopulegol
cis-3-Hexyl isovalerate
Isomenthol
cis-Ocimene
Myrcene
Pulegone
(-)-Carvone
Terpinene-4-ol
gamma-Terpinene
Linalool
3-Octanol
alpha-Humulene
para-Cymene
1-Octen-3-ol
alpha-Terpinene
alpha-Terpineol
cis-Sabinene Hydrate
Terpinolene
Isopulegol
Isovaleraldehyde
3-2-Methyl butanol
0
10
20
30

C

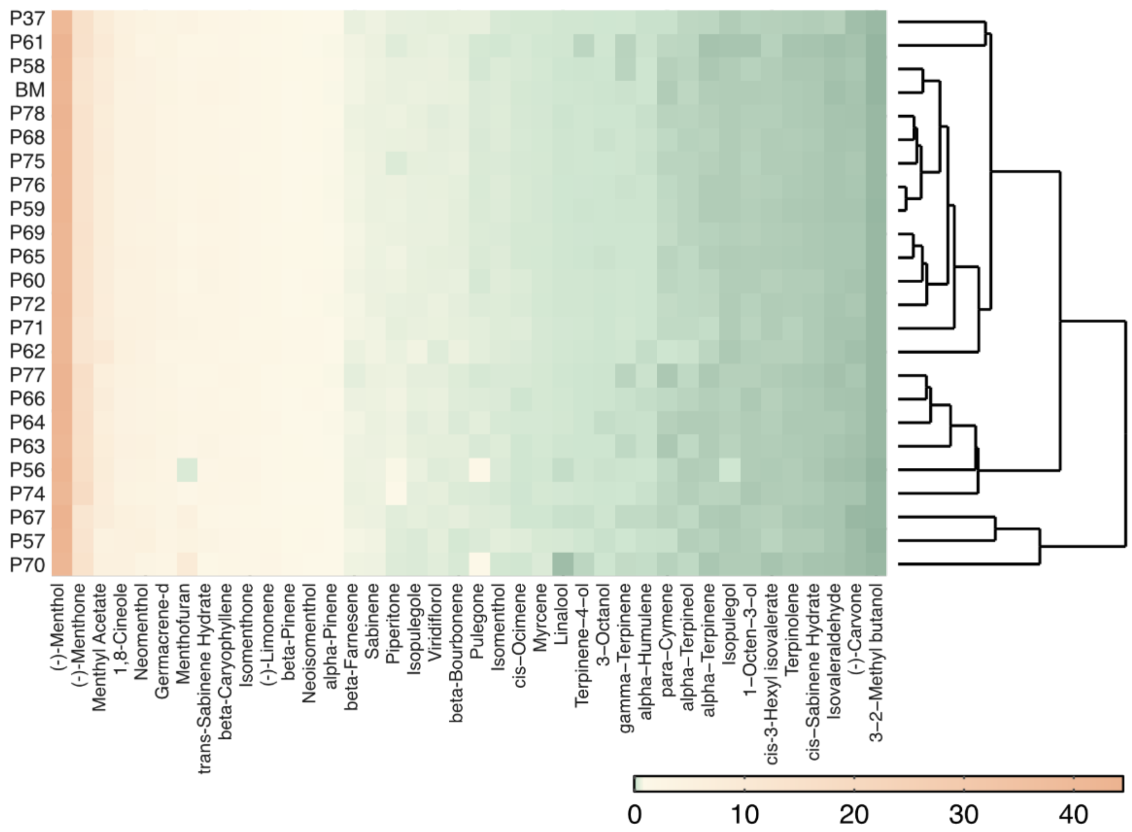

Figure S7. **Oil profiles composition for 3 different sets of mutant lines tested under field conditions.** Colors represent mean values of relative abundance of 3 biological replications. **A**: 2019 field trials. **B**: 2020 field trials. **C**: 2021 field trials. Each year a Black Mitcham control was planted and tested along with the set of mutant lines.

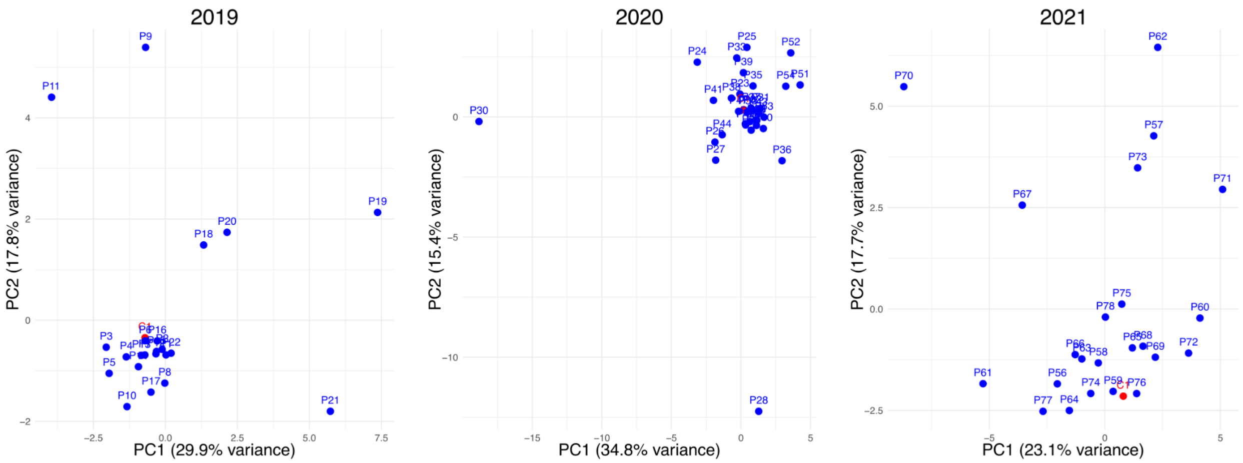

Figure S8: Variation in oil composition under field conditions. Principal component analysis of oil composition from plants in the different seasons. Values are means of three biological replicates. C1: Black Mitcham control (red).

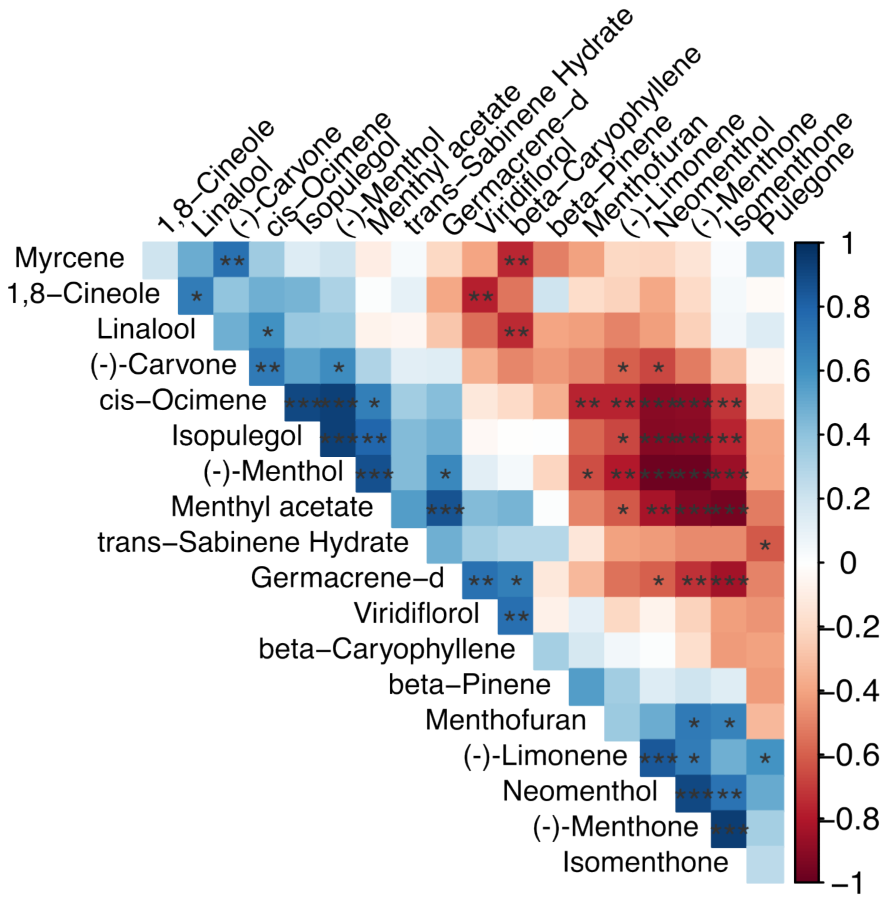

Figure S9. **Correlation plot of oil composition of P11 and P28 mutants along with their controls.** Colors represent positive (blue) and negative (red) correlations. Correlation matrices were visualized using corrplot, with significance indicated at $p < 0.05$, 0.01, and 0.001. *p*-values were obtained from pairwise two-sided Pearson correlation tests between samples.

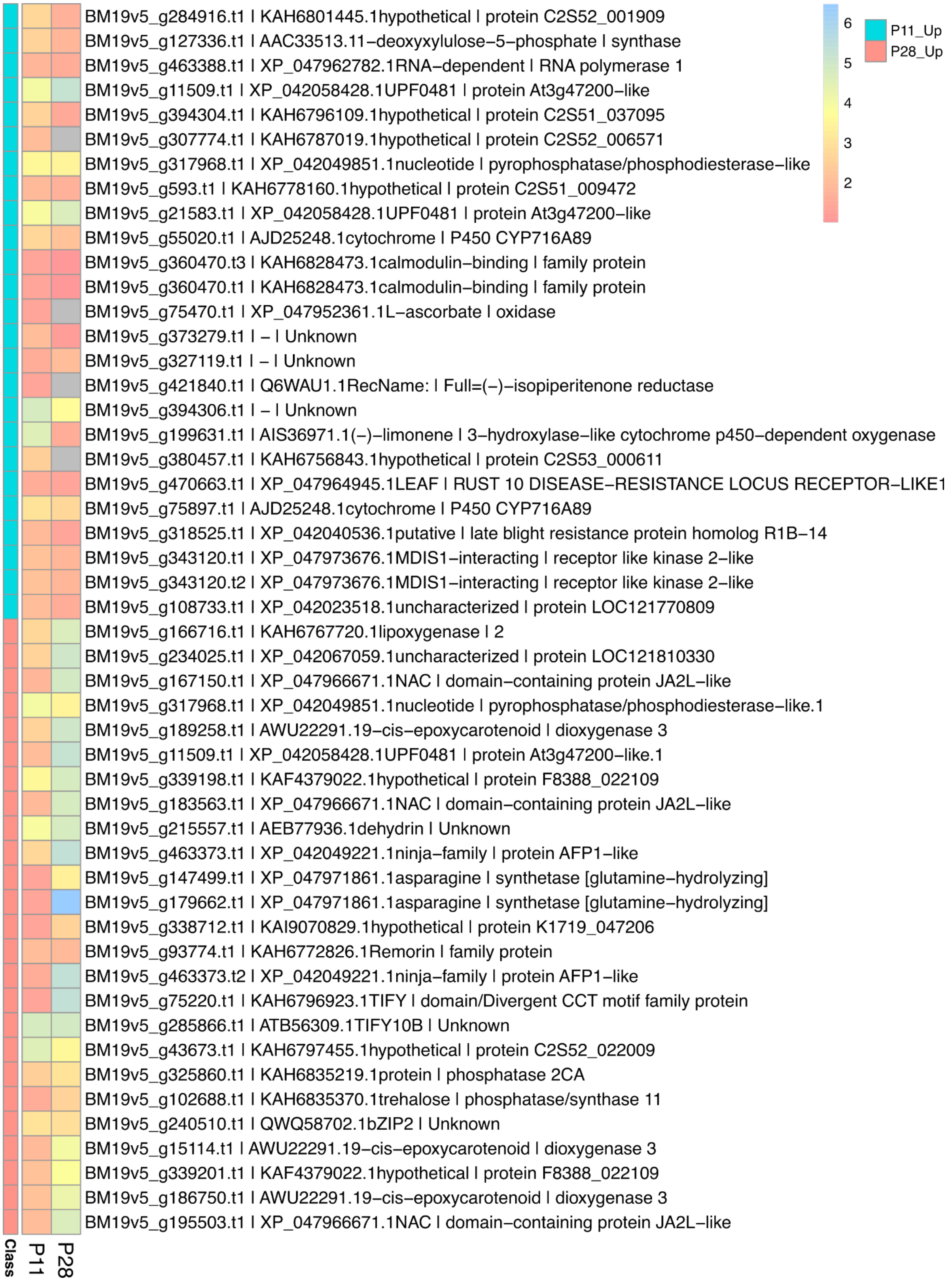

Figure S10. **Upregulated genes in P11 and P28** (Values correspond to log2FoldChange and only Top 25 genes are shown ordered by most significant padj value).

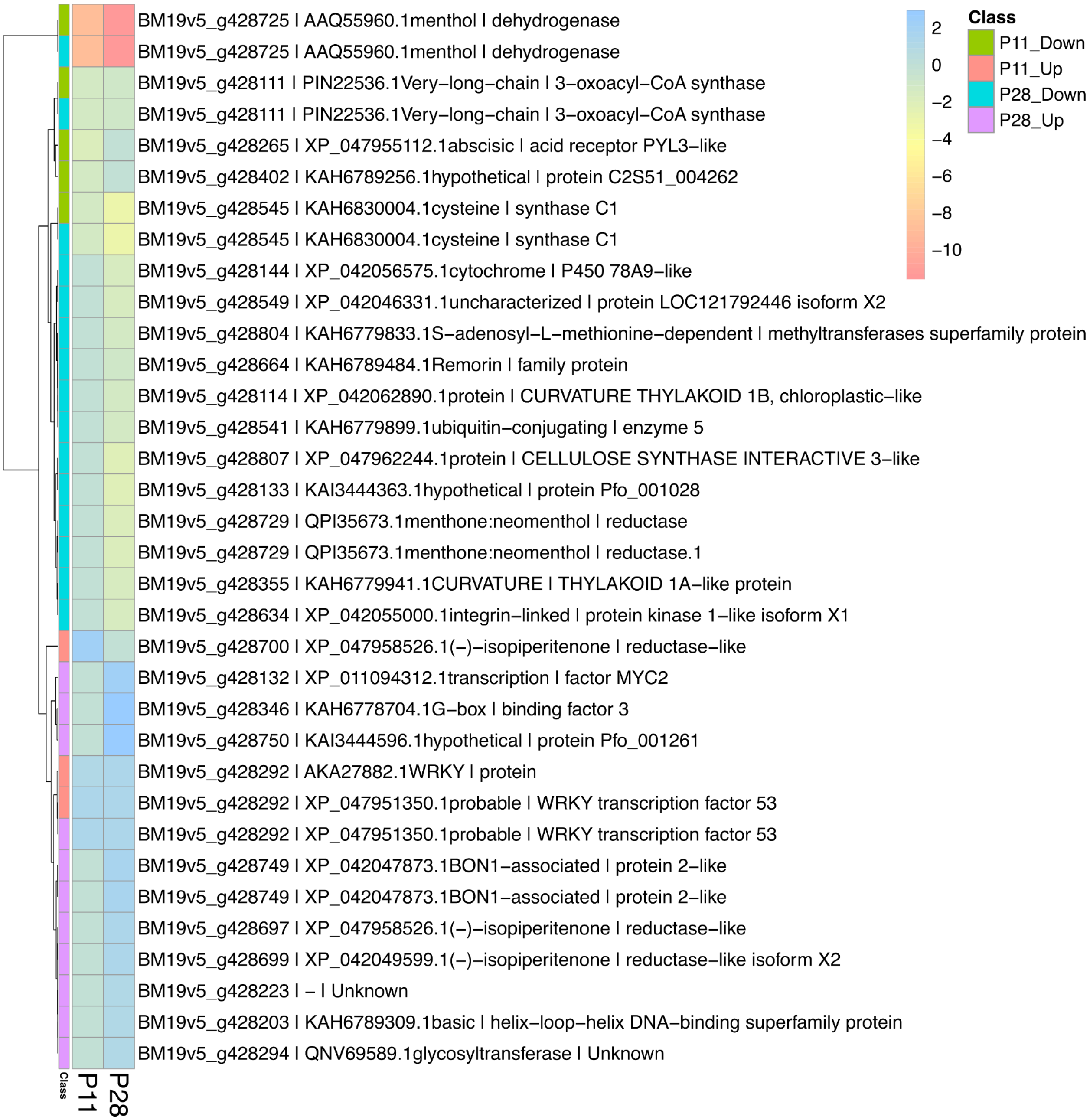

Figure S11. **Differential expression of genes found in the Chr11_D, 0 - 2.5 Mbp region (overlapping indels in both mutants).** Values correspond to differential expressions (log2FoldChange). Note that BM19v5_g428725 is the most downregulated gene from those found in the interval. Duplicated gene IDs on the same gene ID correspond to genes with multiple annotated transcripts (e.g Bm19v5_g428292).

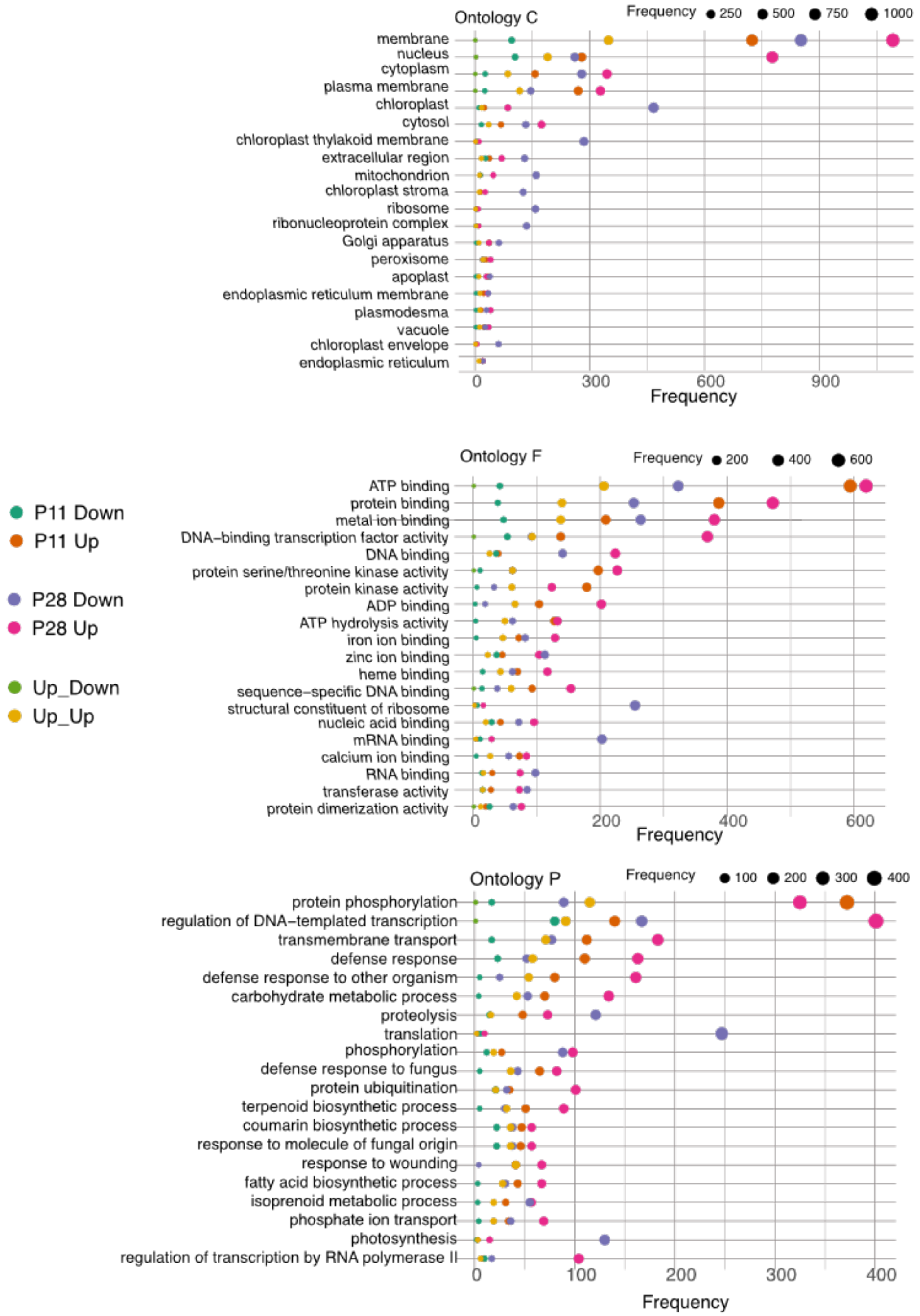

Figure S12. **Functional annotation of differentially regulated genes.** Colors indicate differential regulation direction in one or both low (-)-menthol mutants.

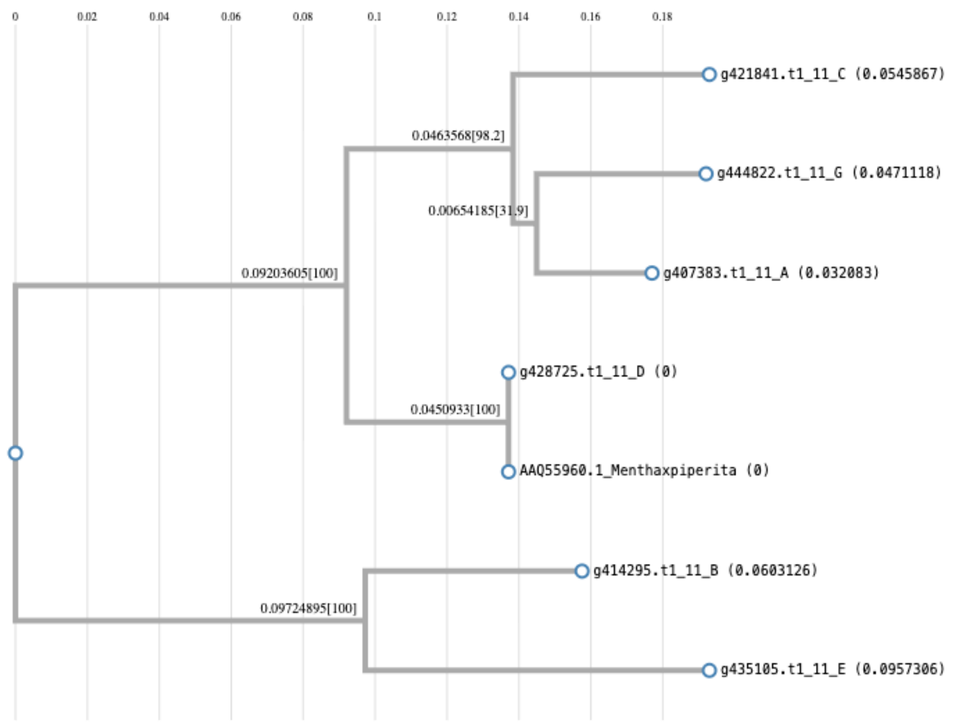

Figure S13. Multiple protein sequence phylogenetic relationships of all copies of MMR in Chr11. A published sequence is used as reference (GenBank: AAQ55960.1). Alignment and phylogenetic reconstructions were performed using the function "build" of ETE3 3.1.3 (Huerta-Cepas et al., 2016) as implemented on the GenomeNet (https://www.genome.jp/tools/ete/).

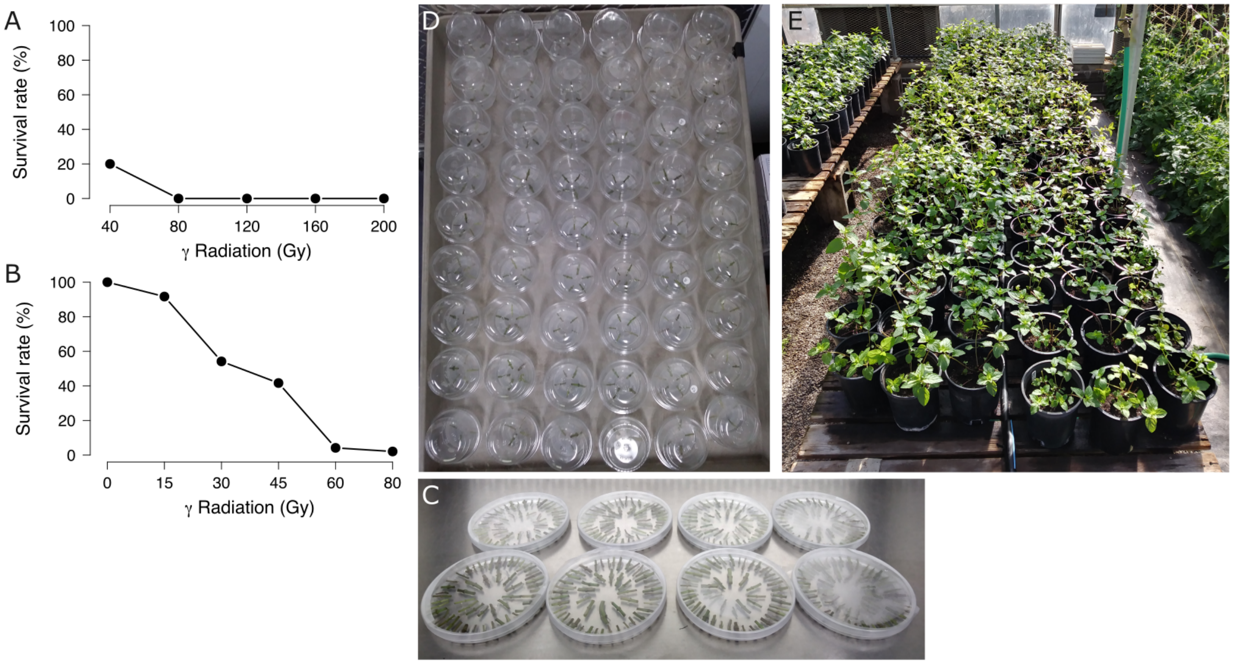

Figure S14. Axillary bud irradiation. (A) First survival test using doses ranging between 40 and 200 Gy. (**B**) Second survival test using doses ranging between 0 and 80 Gy. We found that the 45 Gy dose was optimal to maximize the number of mutations obtained per line, while still recovering 41% of plants, while higher doses presented higher lethality. Plants treated with 45 Gy (**C**) were moved to cups with MS media 4 days after irradiation (**D**). After treatment, plants recovered *in vitro* were transplanted to soil under greenhouse conditions where six cycles of successive propagations were performed, with the goal of obtaining homogeneously mutated clones (**E**).

References

Huerta-Cepas J, Serra F, Bork P. ETE 3: Reconstruction, Analysis, and Visualization of Phylogenomic Data. Mol Biol Evol. 2016 Jun;33(6):1635-8.

Cheng, H., Concepcion, G.T., Feng, X., Zhang, H., Li H. (2021) Haplotype-resolved de novo assembly using phased assembly graphs with hifiasm. Nat Methods, 18:170-175. https://doi.org/10.1038/s41592-020-01056-5

Cheng, H., Jarvis, E.D., Fedrigo, O., Koepfli, K.P., Urban, L., Gemmell, N.J., Li, H. (2022) Haplotype-resolved assembly of diploid genomes without parental data. Nature Biotechnology, 40:1332–1335. https://doi.org/10.1038/s41587-022-01261-x

Cheng, H., Asri, M., Lucas, J., Koren, S., Li, H. (2024) Scalable telomere-to-telomere assembly for diploid and polyploid genomes with double graph. Nat Methods, 21:967-970. https://doi.org/10.1038/s41592-024-02269-8
